# Metabolite profiling of Arabidopsis mutants of lower glycolysis

**DOI:** 10.1038/s41597-022-01673-z

**Published:** 2022-10-11

**Authors:** Youjun Zhang, Alisdair R. Fernie

**Affiliations:** 1 Center of Plant Systems Biology and Biotechnology, 4000 Plovdiv, Bulgaria; 2 Max-Planck-Institut für Molekulare Pflanzenphysiologie, Am Mühlenberg 1, 14476 Potsdam-Golm, Germany

**Keywords:** Plant physiology, Plant molecular biology

## Abstract

We have previously shown that in Arabidopsis the three enzymes of lower glycolysis namely phosphoglycerate mutase (PGAM), enolase and pyruvate kinase form a complex which plays an important role in tethering the mitochondria to the chloroplast. Given that the metabolism of these mutants, the complemented of *pgam* mutant and overexpression lines of PGAM were unclear, here, we present gas chromatography mass spectrometry-based metabolomics data of them alongside their plant growth phenotypes. Compared with wild type, both sugar and amino acid concentration are significantly altered in *phosphoglycerate mutase, enolase* and *pyruvate kinase*. Conversely, overexpression of PGAM could decrease the content of 3PGA, sugar and several amino acids and increase the content of alanine and pyruvate. In addition, the *pgam* mutant could not be fully complemented by either a nuclear target pgam, a side-directed-mutate of pgam or a the *E.coli* PGAM in term of plant phenotype or metabolite profiles, suggesting the low glycolysis complete formation is required to support normal metabolism and growth.

## Abstract (summary)

**Table:** 

Measurement(s)	Metabolite profiling of Arabidopsis mutants of enzymes of lower glycolysis
Technology Type(s)	Gas chromatography–mass spectrometry
Factor Type(s)	Metabolite profiling of lower glycolysis
Sample Characteristic - Organism	Arabidopsis mutants of enzymes of lower glycolysis
Sample Characteristic - Environment	plant grow under 8 h light (22 °C)/16 h dark (18 °C) in growth chamber at a light intensity of 120–150 μmol m-2 s-1
Sample Characteristic - Location	2Max-Planck-Institut für Molekulare Pflanzenphysiologie, Am Mühlenberg 1, 14476 Potsdam-Golm, Germany

## Background & Summary

As one of the hallmark pathways of respiration, glycolysis provides carbon skeletons for the biosynthesis of a wide range of metabolites as well as being at the heart of energy transformations^1,2^. In plants, enzymes of glycolysis exist both in the cytosol and plastid, and thus parallel reactions are catalyzed by differentially localized nuclear-encoded isoenzymes. In plant two ATP-independent network containing alternate cytosolic reactions enhance the pathway’s ATP yield via utilizing pyrophosphate in place of ATP. In the dark, starch is degraded to glucose, and then provides the energy and carbon skeletons by cytosol glycolytic pathway and mitochondrial TCA cycle for the plant growth and development^3^. Chloroplasts and non-photosynthetic plastids use an incomplete glycolytic pathway to breakdown starch as well as to generate carbon skeletons, reductant, and ATP for anabolic pathways such as fatty acid synthesis^1,3^. In the light, 3-phosphoglyceric acid (3PGA) and Glyceraldehyde 3-phosphate (GAP) are produced by photosynthesis and used to synthesize the RuBP and starch in the chloroplast generating ATP either via photophosphorylation or by oxidative phosphorylation to provide energy for metabolic reactions. It has long been reported that GAP and 3PGA are exported to the cytosol by the triose phosphate translocator^4^. This 3PGA could subsequently be converted to pyruvate where after it can be incorporated into the TCA cycle, while GAP could be used to synthesize sucrose or alterative be converted to pyruvate to supply into the TCA cycle^5,6^. In addition, the rate of plant glycolytic flux mostly adjusts to energy and the carbon skeleton demand.

In lower cytosolic glycolysis, phosphoglycerate kinase (PGK) is involved in both photosynthetic carbon metabolism and glycolysis/gluconeogenesis^7^. Phosphoglycerate kinase (PGK) could catalyze the reversible reaction transferring a phosphate group from 1,3-bisphosphoglycerate (1,3-BPG) to ADP producing 3PGA and ATP^1^. Indeed, the cytosolic *pgk* knockout mutant was characterized as having reduced growth but higher starch levels than the wild type^7^. In addition, the single phosphate group left on the 3PGA could be transferred to central carbon by 2,3-biphosphoglycerate-independent phosphoglycerate mutase (PGAM) to produce 2-phosphoglycerate (2-PGA). Although single *pgam* mutants were similar to the wild type in all plant phenotypes assayed, double mutants had no detectable PGAM activity and showed defects in abscisic acid-, blue light-, and low CO_2_-regulated stomatal movement^8^. Vegetative plant growth was also severely impaired in the double mutants and no pollen was produced^8^. The subsequent step of the pathway that is catalyzed by enolases result in the reversible dehydration of 2-PGA to phospho*enol*pyruvate (PEP) which is required for both ATP production and aromatic compound and secondary metabolite biosynthesis^9^. Interestingly, cytosolic enolase encodes two proteins namely a full-length enolase and a truncated version cMyc binding protein (AtMBP-1) which is a nuclear localized transcript factor^10^. The single *enolase* mutant displays several cellular defects including reduced cell size, defective cell differentiation with restricted lignification, as well as, altered vascular development, impaired floral organogenesis and defective male gametophyte function, resulting in embryo lethality^9^. The last enzyme of the glycolysis is pyruvate kinase which is important not only in ATP production but also in providing carbon skeleton for fatty acid biosynthesis. In Arabidopsis, five identified cytosolic pyruvate kinase isoforms adjust the carbohydrate flux through the glycolytic pathway^11^. Both PKC3 and PKC4 are dual localized to the cytosol and mitochondrial outer membrane but mutants of all isoforms have not yet been studied. Although the plant growth phenotype and physiology of the remaining lower glycolytic mutants were well investigated, the metabolic consequences of these genetic interventions, with the exception of the *pgk* mutant, have not been well characterized.

In our previous research, the glycolytic complex of PGAM, enolase and pyruvate kinase was found to not only be involved in substrate channeling but also have a moonlighting role in mediating the co-localization of mitochondria and chloroplasts^2^. Two studies suggested that PGAM and enolase are formed a metabolon which efficiently convers 3PGA to 2PGA at the outer membrane of the mitochondria. Given that the double mutant of the *pgam* displayed greatly inhibited growth, it is important to understand the metabolic function of the metabolon by complementing *pgam* mutants using *E.coli* homologs, as well as non-functional and nuclear targeted PGAMs. In addition, given that the overexpression of the PGAM1 resulted in slightly increase in plant growth, it is important to evaluate the metabolite composition. Here, the primary metabolism of all the low glycolytic enzymes mutants, PGAM overexpression and complementation lines were analyzed by the GC-MS according to our recently method to provide data set for cross-study comparisons of plant metabolites^12^, investigations into the reproducibility of metabolomics data, in-depth analysis of plant metabolism and mathematical modelling of glycolytic flux.

## Methods

### Plant growth conditions

*Arabidopsis thaliana* genotypes Columbia (Col-0) (WT), *pgk* (SALK_123919), *pgam1-1* (SALK_003321), *pgam 1-2* (SALK_029822), *pgam 2-2* (SALK_002280), *pgam1/2* (SALK_029822/SALK_002280), *enolase2*–*4* (SAIL_208_E09), *pkc3* (GABI_187A04) and *pkc4* (SALK_143658) mutants, PGAM1 overexpression lines and *pgam1/2* complementation lines (*nA- pgam1/2-1*, (nuclear target PGAM1), *sdmA-pgam1/2 -1*, (non-functional PGAM1), *E.pgam-pgam1/2 -1 (E.coli* PGAM), *sdmA-E.pgam-pgam1/2 -1 (E.coli* PGAM and native promoter PGAM1 with nonfunctional PGAM1) and *pgam-pgam1/2* (native PGAM1), Supplementary Table 1) were used in this study. The seeds were plated on Murashige and Skoog medium supplemented with 1% (w/v) sucrose for 10 days, then the seedlings were transferred to soil under 8 h light (22 °C)/16 h dark (18 °C) period (short day) in growth chamber at a light intensity of 120–150 μmol m^−2^ s^−1^. The PGAM mutant and the overexpression lines were also transferred to 16 h light (22 °C)/8 h dark (18 °C) (long day) in growth chamber at a light intensity of 120–150 μmol m^−2^ s^−1^ for plant phenotype. The pre-bolting mature rosette leaves of 35-day-old short day *Arabidopsis* were harvested for metabolite measurement after two hours of light (10 am).

### Cloning genes of the PGAM

The cytosolically localized PGAM1, PGAM1 with promoter and terminator, E.coli PGAM and enolase promoter were amplified and subcloned following previously published protocol^13^. These genes sub-cloned into gateway entry vectors the pDnor207 donor vector by gene specific primers (Supplementary Table 2). The activity site of PGAM1 was mutated by five amino acid (H39, S80, K367, DH470) as primer Supplementary Table 3. Expression vectors for overexpression and complementation were constructed using the Gateway LR reaction with pK7WG2 and PMDC110^14^ (Supplementary Table 3).

### Plant transformation

These constructs were transformed into the *Agrobacterium tumefaciens* strain GV3101 and then transformed by the floral dip method^15^ into wild type plants or the *pgam1/2* double mutant by the green team of the Max Planck Institute of Molecular Plant Physiology. Homozygous overexpression (OE) lines and complementation were selected on MS plates containing 50 mg/L Kanamycin or 100 mg/L Hygromycin. Resistant lines were transferred to soil to grow to maturity, and their transgenic status was confirmed by genomic PCR. Homozygous and complementation T3 seeds of the OE plants were used for further analysis.

### Metabolite measurement

Metabolite profiling of *Arabidopsis* leaves was carried out by gas chromatography–mass spectrometry (ChromaTOF software, Pegasus driver 1.61; LECO) as described previously^16,17^. Briefly, around 50 mg plant materials were frozen and extracted in 700 µl 100% methanol and mixed with 30 µl ribitol (0.2 mg/ml ddH_2_O) at room temperature by vortexing. After centrifugation (20000 g, 10 mins), the upper phase was subsequently extracted in 375 µl chloroform and 750 µl ddH_2_O. After centrifugation (20000 *g*, 5 mins) the 150 µl upper phase was dried by speed vac for the GC-MS measurement^18,19^. Samples were derivatized using the standard protocol^17^. Briefly, 40 μl Methoxyaminhydrochlorid (20 mg/ml in Pyridin) was used to resuspend the metabolites with 2 h shaking at 37 °C. 70 μl MSTFA (1 mL MSTFA + 20μL FAME mix) was added with other 30 mins shark at 37 °C. The supernatant was transferred to GC sample vials. The chromatograms and mass spectra were evaluated using ChromaTOF software. Metabolite identification was manually checked by the mass spectral and retention index collection of the Golm Metabolome Database^20^. Peak heights of the mass fragments were normalized on the basis of the fresh weight of the sample and the added amount of an internal standard (Ribitol). Statistical differences between groups were analyzed by Student’s *t*-tests on the raw data. Results were determined to be statistically different at a probability level of P < 0.05. Relative metabolite levels were obtained as the ratio between the lines and the mean value of the respective wild type (Supplementary Tables 4, 5 and 6). This method is related to our standard protocol^12^. Principal component analysis (PCA) was performed using MetaboAnalyst (https://www.metaboanalyst.ca/).

## Data Records

Raw data of GC-MS have been deposited in the Metabolights^21^ (MTBLS4066^22^: Metabolite profiling of Arabidopsis mutants of lower glycolysis) with Arabidopsis wild-type (Col-O), eight mutants, three overexpression and six complementation lines. Raw data contained quality control with 41 metabolites as standard for data analysis. Peg files contain ion detections without data of in-source fragmentation using collision energy. This dataset was composed by two parts of measurement. The first part is the seven glycolytic mutants, three overexpression lines and one wild-type each with six biological replicates. It had 61 metabolites which could be easily characterized (Supplementary Tables 4 and 5, detail analysis at metabolights raw1).The second part is the ipgam1/2 complementation lines with double mutant and WT with six biological replicates. All the metabolite data were analyzed by the ChromaTOF software and searched on the Golm Metabolome Database as mentioned above. The second part contained 48 well characterized metabolites (Supplementary Table 6, detail analysis at metabolights raw2). The details of the data analysis upload to metabolights as raw1 and raw2.

## Technical Validation

The standard deviation was calculated to qualitatively validate metabolite data obtained from at least four biological samplesas the square root of variance by determining each data point’s deviation relative to the mean. Metabolites were qualitatively validated either by forty-one chemical standards (QC) or by Arabidopsis controls (Supplementary Table 7).

## Usage Notes

### Plant phenotype and metabolite profiles of pgk, phosphoglycerate mutase, phosphoglycerate mutase 2, enolase, pkc3, pkc4 mutants

**Fig. 1:**
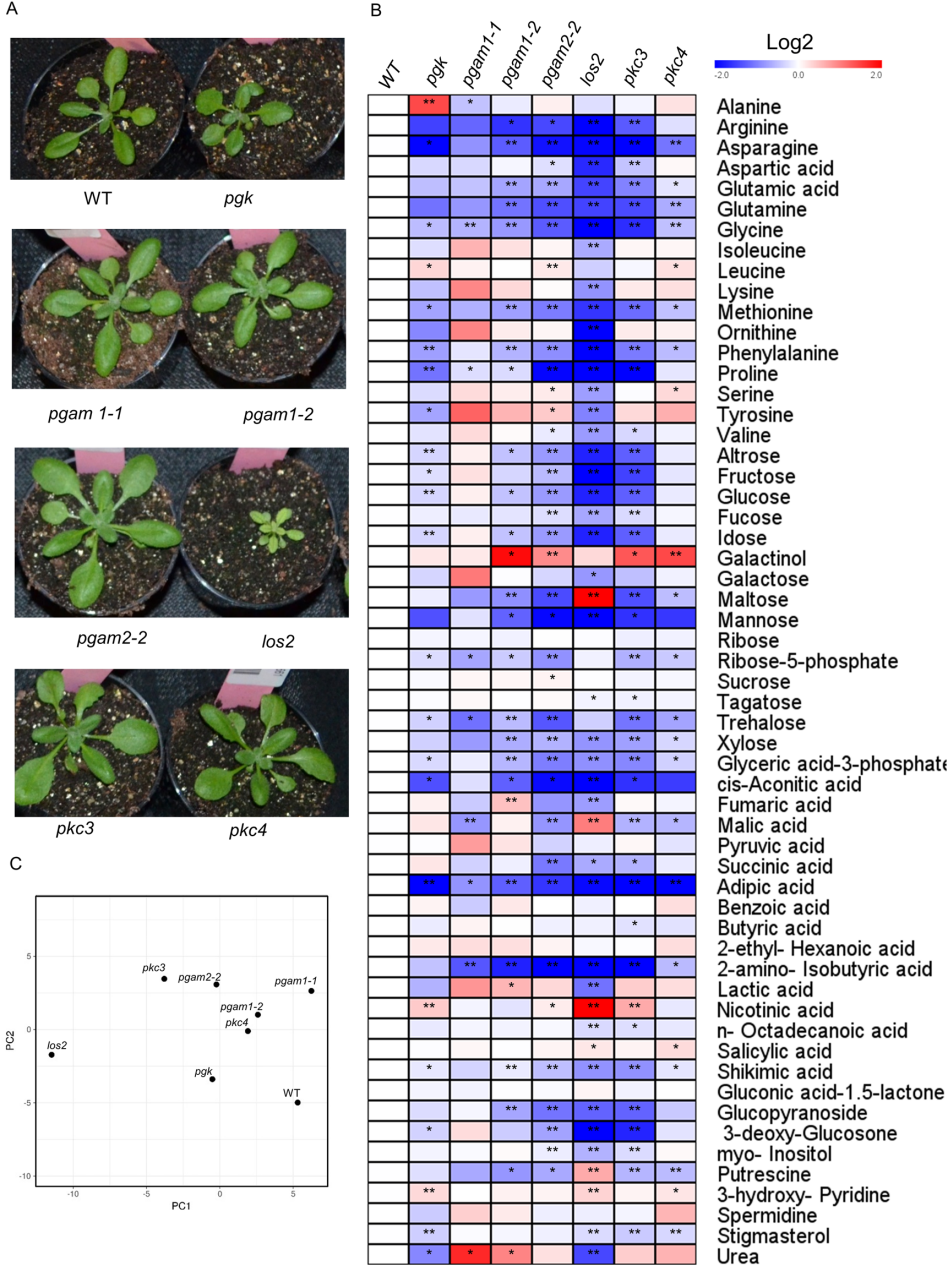
Plant growth phenotype and metabolite profiling of Arabidopsis mutants. (**A**) The plant phenotype of 35-day-old plant grew in the green house under short day conditions (8 h light and 16 h dark). Similar to the published growth deficient of enolase^9^, the pgk mutant also presented slow growth compared with the wild type, while the other mutant did not show a different growth phenotype. (**B**) Metabolite profiling of mutants presented as a heat map calculated by log2 fold change. 30 day-old-plant leaves of SD condition were collected at the 10 am and measured the metabolites by GC-MS (*phosphoglycerate kinase* (*pgk*), *phosphoglycerate mutase 1-1* (*pgam1-1*), *phosphoglycerate mutase 1-2* (*pgam1-2*)*, phosphoglycerate mutase 2-2* (*pgam2-2*)*, enolase (los2), pyruvate kinase-3* (*pkc3*) and *pyruvate kinase-4* (*pkc4*)). (**C**) PCA analyse the metabolism.

Given that the phosphoglycycerate mutase, enolase and pyruvate kinase complex plays an important role in plant growth, 3PGA metabolism and the chloroplast mitochondria association, both the plant growth phenotype and metabolite profiling analysis were carried out on the respective mutant lines (Fig. 1A). Given that the PGK can also use 3PGA, the mutant of *pgk* was selected and demonstrated to grow more slowly compared with the wild type. The levels of several amino acids and sugars were significantly changed compared with WT plant (Fig. 1B). The plant growth phenotypes of *phosphoglycerate mutase 1* and *phosphoglycerate mutase 2* have been published^8^, but the metabolic changes occuring in these mutant remains unclear. The *phosphoglycerate mutase 1* mutant exhibited decreased contents of glycine and glucose, while *phosphoglycerate mutase 1* mutant significantly decreased several amino acids and fructose and glucose. The *enolase* mutant, however, was characterized by reduced shoot and root growth, altered vascular development and defective secondary growth of stems, impaired floral organogenesis and defective male gametophyte function, resulting in embryo lethality as well as delayed senescence. In our metabolite analysis, the *enolase* mutant contained low amount of amino acids and sucrose, indicating an important role of sucrose synthesis and glycolysis (Fig. 1B and C). The mutant of *pkc3* and *pkc4* neither showed the significant phenotypic changes following growth on 1% glucose medium or on soil. Metabolic profiling of this mutant revealed that galactinol and urea were increased, while mannose, aconitic acid, adipic acid and several amino acids were decreased (Fig. 1B). However, the biological significance of these changes remains unclear.

### Plant growth phenotype and metabolite profiles of phosphoglycerate mutase 1overexpression and mutant

**Fig. 2:**
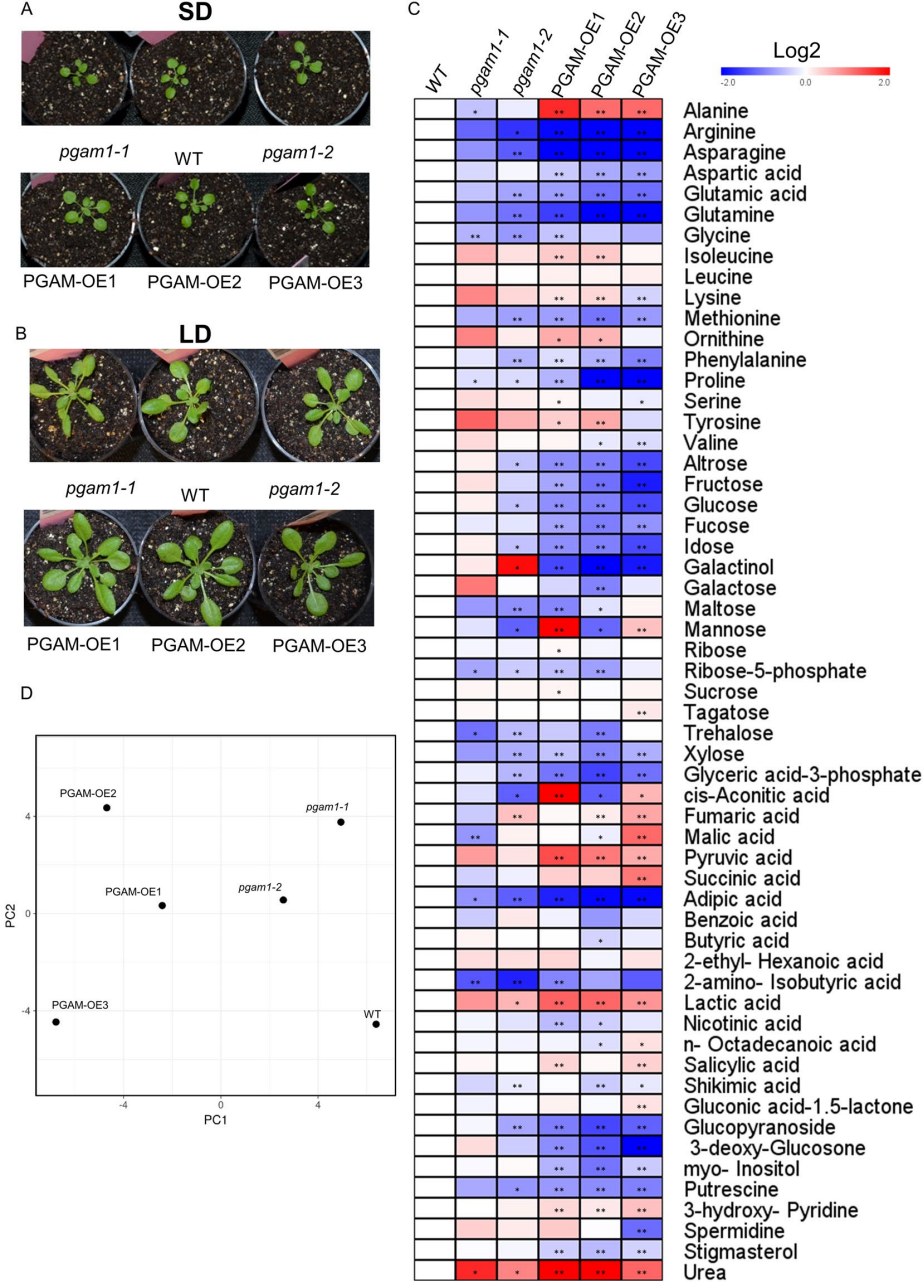
Plant growth phenotype of and metabolite profiling of PGAM mutant and the overexpression lines. 28 days plants in the short day (SD) (**A**) and 28 days plants in the long day (LD) condition (**B**). The phosphoglycerate mutase 1 OE lines grew faster in the LD condition of low light condition while there was no significant difference in the normal condition. (**C**) Metabolite profiling of mutant and overexpression lines in the heat map calculated by log2 fold change. 35 days old plant leaves of SD condition were collected at the 10 am and measured the metabolites by GC-MS. (**D**) PCA analyse the metabolism.

Given the presence of the lower glycolysis metabolon of PGAM, enolase and pyruvate kinase likely plays an important role in the metabolite exchange of chloroplast and mitochondria^2^, phosphoglycerate mutase 1 was overexpressed under the control of the constitutive 35 S promoter (Fig. 2). The plant growth phenotype was neither significantly altered in short or long day, while the overexpression lines grew faster in the low light condition with four~six more leaves per plant (Fig. 2A). In the metabolite profiling analysis of normal light condition, the OE lines had lower content of 3PGA, glucose, frucose, arginine, asparagine, glutamine, proline, galactinol and adipic acid (Fig. 2B,C). By contrast, the content of alanine and pyruvate acid were significantly increased in the OE lines.

### Complementation of the metabolic and morphological phenotypes

In our former research, the physical interaction between the mitochondria and chloroplast appears to be greatly influenced by the phosphoglycerate mutase-enolase-pyruvate kinase association which we demonstrate above has the capacity to very highly efficiently convert 3PGA to pyruvate. In order to further study the importance of the constituent enzymes in the co-localization of mitochondria and chloroplasts we studied the phosphoglycerate mutase double mutant and various complemented versions of these mutants at the metabolic, morphological and cell biological levels. The mutants could be fully complemented at both the enzyme activity and plant morphology levels following the expression of the corresponding gene under the control of its native promoter^8,9^. In addition we alternatively attempted to complement the *pgam* double mutant with the full length PGAM targeted to the nucleus, with a site-directed-mutant effecting a residue of the active site of PGAM1 and thus being catalytically inactive^8,23^, or with the *E. coli* PGAM. In the nuclear sublocalized PGAM1 complementation lines, the enzyme activity could be partial complemented to levels resembling those previously reported for the single *pgam* mutants^8^, by contrast the plant growth and development could not recovered and the complemented lines still produced less seeds. In addition, the side-directed-mutated Arabidopsis PGAM could neither complement the enzyme activity nor the plant growth and developmental phenotypes^1^. The *E.coli* PGAM did not interact with enolase or TPT and could only recover 50% enzyme activity, it also neither complemented seed production nor seed growth^17^.

**Fig. 3:**
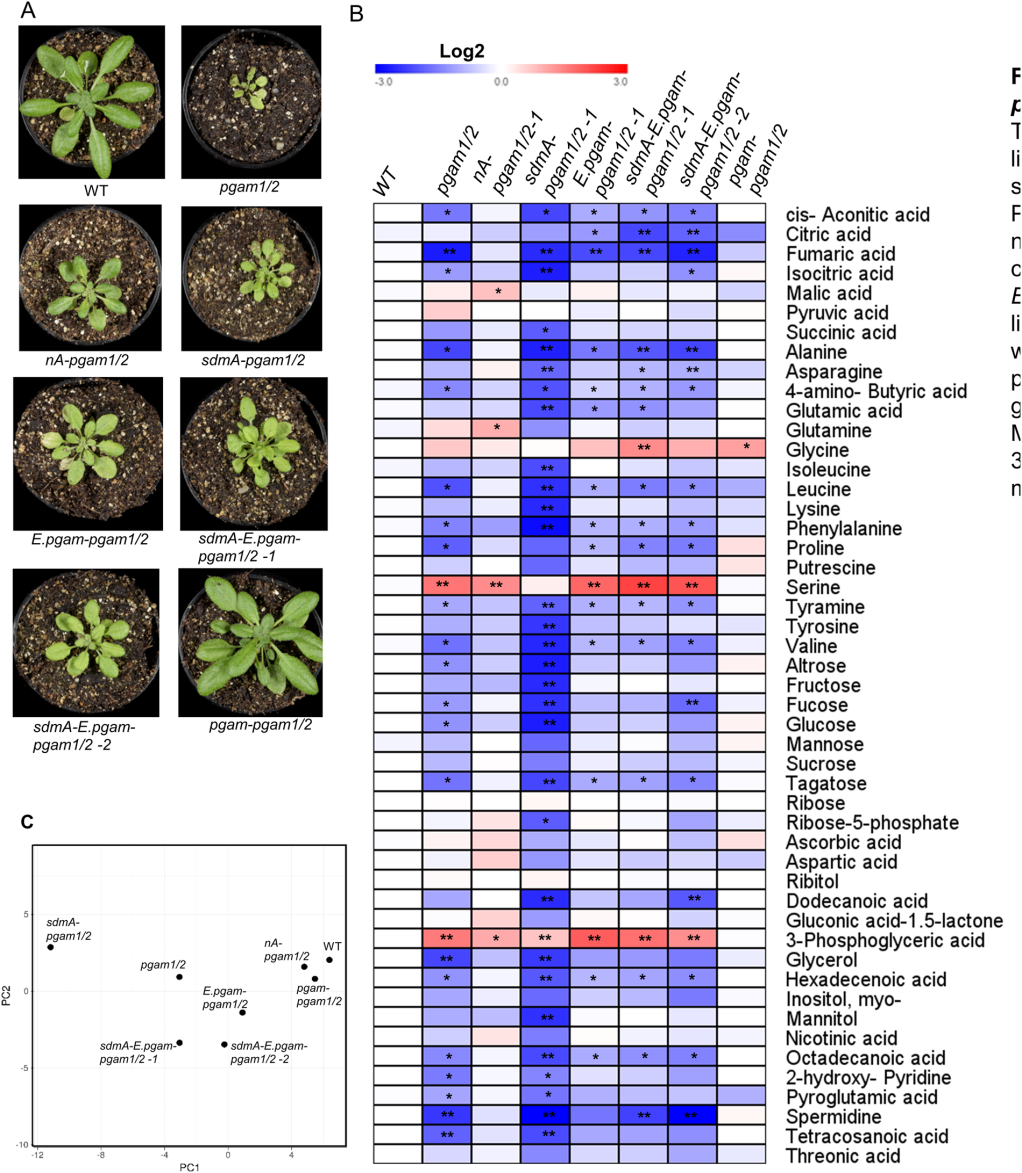
Plant growth phenotype and metabolite profiling of *phosphoglycerate mutase* double mutant complement lines *and WT*. (**A**) The plant phenotype of 35-day-old plant grew in the green house with short day condition (8 h light and 16 h dark). The double mutant of *phosphoglycerate mutase* is very small in the soil. *nA- pgam1/2-1* and *nA- pgam1/2-2 are* two complementation lines native promoter PGAM1 with nuclear target PGAM1. *sdmA-pgam1/2 -1* and *sdmA-pgam1/2 -2* are two complementation lines native promoter PGAM1 with nonfunctional PGAM1. *E.pgam-pgam1/2 -1* and *E.pgam-pgam1/2 -2* are two complementation lines native promoter enolase with *E.coli* PGAM. *sdmA-E.pgam-pgam1/2 -2* and *sdmA-E.pgam-pgam1/2 -2* are two complementation lines native promoter enolase with *E.coli* PGAM and native promoter PGAM1 with nonfunctional PGAM1. *pgam-pgam1/2* is the complementation lines native promoter PGAM1 and full length PGAM1. *nA- enolase-2* is the enolase-2 complemented by native promoter with nuclear target enolase. All the complemented lines presented growth slowly compared with the wild type and full completed lines. (**B**) Metabolite profiling presented in the heat map calculated by log2 fold change. 35 days old plant leaves of SD condition were collected at 10 am and measured the metabolites by GC-MS. (**C**) PCA analysis the metabolism.

Having characterized visiable phenotype, we next performed metabolite profiling on the double *pgam* mutants and a number of complementation lines being able to identify 49 primary metabolites using gas chromatography–mass spectrometry (GC-MS) (Fig. 3). In the double mutant, several intermediates of the TCA cycle (fumarate, aconitic acid and isocitric acid) and several amino acids (alanine, leucine, phenylalanine and valine) were significantly decreased. However, 3PGA, glycine and serine were increased indicating the importance of the PGAM reaction within the regulation of carbon partitioning. These changes were essentially reverted on the complementation of the PGAM by the expression of PGAM under the control of its native promoter. However, as would perhaps be expected following expression of PGAM in the nucleus had little effect on the metabolome of the double mutant and neither did expression of the catalytically inactive PGAM. Similarly, the ectopic expression of the *E. coli* PGAM under the control of plant enolase native promoter could not complement the mutant metabolome.

## Supplementary information

Supplementary table

## Data Availability

All code used in this study has been deposited with the data at MetaboLights MTBLS4066^22^: Metabolite profiling of Arabidopsis mutants of lower glycolysis.
