# Supplementary material for: Metabolite profiling of Arabidopsis mutants of lower glycolysis
Source: Sci Data. 2022 Oct 11;9:614. doi: 10.1038/s41597-022-01673-z (PMC9553893; doi:10.1038/s41597-022-01673-z)
Supplement: Supplementary file 1 — Supplementary table [file 41597_2022_1673_MOESM1_ESM.docx]

| **Supplementary table1a Mutants and overexpression lines used in this study** | | | | | |
| --- | --- | --- | --- | --- | --- |
| AGI |  | mutant and overexpression lines | LP-primer | RP primer | Provider |
| At1g79550 | *pgk* | SALK_123919 | TACCTGAAGGTGGTGTTTTGC | CAGCAACAGAGTCACCTCCTC | NASC |
| At1g09780 | *phosphoglycerate mutase (pgam) 1-1* | SALK_003321 | ATGTTCTATCAATCTCCGGGG | TCTGCCCCTGATCAGTACAAC | Prof. Sarah M. Assmann，Penn State University, USA (Zhao and Assmann, 2011) |
|  | *pgam 1-2* | SALK_029822 | CCATATCAAGCGGTGAAGTTC | ATCTGAGCATCCACACCATTC |  |
| AT3G08590 | *pgam 2-2* | SALK_002280 | ATTGTCTGCAAACCAGGACAC | TTAAAAGCGCTCTTGAAGCAG |  |
| At1g09780/ AT3G08590 | *pgam1/2* | SALK_029822/ SALK_002280 |  |  |  |
| At2g36530 | *enolase2-4* | SAIL_208_E09 | TGCCGCTTCAGAGTTCTACTC | GCAGAGCATTCTCATGTTTCC | Prof. Brigitte Poppenberger，Technische University Muenchen (Eremina et al., 2015) |
| At5g63680 | *pkc3* | GABI_187A04 | CGCTGACTTCAAAGCAGATTC | ATTATGACATCAAGGGCGATG | Dr. Stephan Krueger (University of Cologne) |
| AT5G08570 | *pkc4* | SALK_143658 | GAAATTCCCAAAGAACCGAAG | GCTTGCAAAAGGAAACACAAG |  |
| At1g09780 | PGAM OE1 | Overexpression |  |  | Max Planck Institute of Molecular Plant Physiology |
|  | PGAM OE2 | Overexpression |  |  |  |
|  | PGAM OE3 | Overexpression |  |  |  |
|  |  |  |  |  |  |
| **Supplementary table1b *pgam1/2* complementation lines** | | | | | |
|  | Mutant |  |  |  |  |
| nA- pgam1/2-1 | *pgam1/2* | SALK_029822/ SALK_002280 | native promoter PGAM1 with nuclear target PGAM1 | |  |
| sdmA-pgam1/2 -1 | *pgam1/2* | SALK_029822/ SALK_002280 | native promoter PGAM1 with nonfunctional PGAM1 | |  |
| E.pgam-pgam1/2 -1 | *pgam1/2* | SALK_029822/ SALK_002280 | native promoter enolase with E.coli PGAM | |  |
| sdmA-E.pgam-pgam1/2 -1 | *pgam1/2* | SALK_029822/ SALK_002280 | native promoter enolase with E.coli PGAM and native promoter PGAM1 with nonfunctional PGAM1 | |  |
| sdmA-E.pgam-pgam1/2 -2 | *pgam1/2* | SALK_029822/ SALK_002280 | native promoter enolase with E.coli PGAM and native promoter PGAM1 with nonfunctional PGAM1 | |  |
| pgam-pgam1/2 | *pgam1/2* | SALK_029822/ SALK_002280 | native promoter PGAM1 and full length PGAM1 | |  |

**Supplementary table2 primers used for this research**

| Pro-PGM1-f | AAAAAAGCAGGCTCCACCCATCACCTGGCCAACACCACgtaactcttcttcc |
| --- | --- |
| PGAM-f | AAAAAAGCAGGCTCCACCATGGCTACCTCCTCCGCTT |
| PGAM-str | CAAGAAAGCTGGGTcctaCTCCACTACTTCAATCAGGGTGGGC |
| PGAM-nlsr | CAAGAAAGCTGGGTcctaGATGTCGAGTCCAGCCAACTTCAGAGCTAGCTCCACTACTTCAATCAGGGTGGGC |
| pgamH39f | gtggagttGCctctcgtctcgatcaactac |
| pgamH39r | gagacgagagGCaactccaccgtcactgag |
| pgamS80f | tgggaaacGCtgaggttggtcataatgctc |
| pgamS80r | accaacctcaGCgtttcccatatcatcttc |
| pgamK360f | agaccgtcGC**g**tttgggcatgtcaccttct |
| pgamK360r | atgcccaaacGCgacggtctcactgcaagc |
| pgamDH470f | gactgct**gCtGCc**ggaaacgcagaggacatggtg |
| pgamDH470r | cgtttccgGCaGcagcagtcacaacataaattcc |
| PenoE.pgam-f | tttctctagatctactcgctATGTTGGTTTCTAAAAAACCTATGGT |
| TenoE.pgam-r | gcttctaaaagctccatttaTTATTCCACGATGAACAGCGG |
| E.pgam-f | AAAAAAGCAGGCTCCACCATGTTGGTTTCTAAAAAACCTATGGT |
| E.pgam-r | CAAGAAAGCTGGGTcTTCCACGATGAACAGCGGCTT |
| AttB1adapter | GGGGACAAGTTTGTACAAAAAAGCAGGCT |
| AttB2adapter | GGGGACCACTTTGTACAAGAAAGCTGGGT |

**Supplementary table3 construct used for this research**

| Donor | Degestion vector | Function |
| --- | --- | --- |
| 207-PGAM1 | PK7wg2 | PGAM overexpression with 35s promoter |
| 207-Pro-PGAM | pmDC110 | PGAM complementary with native promoter |
| 207-proenolase-E.pgam | pmDC110 | E.coli PGAM complementary by enolase native promoter |
| 207-PGAM mutant (H39,S80,K360,H470) | pmDC110 | mutant activity site PGAM complementary by native promoter |
| 207-pro-PGAMnls | pmDC110 | nulcear tarted PGAM complementary with native promoter |

**Supplementary table4 metabolites normalized by WT of low glycolysis mutants**

|  | WT1 | WT2 | WT3 | WT4 | WT5 | WT6 | PGK-1 | PGK-2 | PGK-3 | PGK-4 | PGK-5 |  | PGK-6 | IPGAM1-1-1 | IPGAM1-1-2 | IPGAM1-1-3 | IPGAM1-1-4 | IPGAM1-1-5 | IPGAM1-1-6 | IPGAM1-2-1 | IPGAM1-2-2 | IPGAM1-2-3 | IPGAM1-2-4 | IPGAM1-2-5 | IPGAM1-2-6 |
| --- | --- | --- | --- | --- | --- | --- | --- | --- | --- | --- | --- | --- | --- | --- | --- | --- | --- | --- | --- | --- | --- | --- | --- | --- | --- |
| Aconitic acid, cis- | 1.85 | 1.37 | 1.09 | 0.91 | 0.31 | 0.34 | 1.68 | 0.67 | 0.38 | 0.38 | 0.31 |  | 0.30 | 0.16 | 0.16 | 0.80 | 0.87 | 0.97 | 0.99 | 0.43 | 0.54 | 0.36 | 0.62 | 0.34 | 0.42 |
| Adipic acid | 1.79 | 1.34 | 1.12 | 0.88 | 0.81 | 0.75 | 0.19 | 0.19 | 0.20 | 0.23 | 0.21 |  | 0.17 | 0.55 | 0.54 | 0.75 | 0.72 | 0.56 | 0.51 | 0.46 | 0.39 | 0.44 | 0.51 | 0.36 | 0.36 |
| Alanine, DL- | 1.38 | 1.30 | 1.08 | 0.82 | 0.92 | 0.90 | 2.60 | 2.15 | 2.97 | 3.03 | 2.78 |  | 2.47 | 0.23 | 0.27 | 0.72 | 0.78 | 0.72 | 0.74 | 0.62 | 0.67 | 0.87 | 0.97 | 0.92 | 1.08 |
| Altrose, D- | 1.07 | 1.09 | 1.02 | 0.96 | 0.98 | 0.95 | 0.80 | 0.78 | 0.83 | 0.82 | 0.79 |  | 0.80 | 0.51 | 0.51 | 1.15 | 1.11 | 1.09 | 1.10 | 1.06 | 1.04 | 0.72 | 0.72 | 0.55 | 0.55 |
| Arginine, DL-, -NH3 | 1.03 | 0.86 | 0.67 | 0.97 | 2.12 | 2.35 | 0.32 | 0.35 | 2.03 | 2.10 | 0.33 |  | 0.37 | 0.34 | 0.31 | 1.45 | 1.33 | 0.43 | 0.44 | 0.38 | 0.40 | 0.35 | 0.31 | 0.17 | 0.18 |
| Asparagine, DL- | 1.05 | 0.92 | 0.73 | 0.95 | 1.51 | 1.62 | 0.25 | 0.25 | 0.79 | 0.89 | 0.26 |  | 0.24 | 0.49 | 0.43 | 1.14 | 1.09 | 0.55 | 0.56 | 0.42 | 0.41 | 0.46 | 0.41 | 0.26 | 0.25 |
| Aspartic acid, L- | 1.20 | 0.97 | 0.82 | 0.81 | 1.03 | 1.12 | 0.80 | 0.78 | 1.29 | 1.41 | 0.82 |  | 0.79 | 0.60 | 0.60 | 1.71 | 1.73 | 0.80 | 0.81 | 0.70 | 0.73 | 0.94 | 0.96 | 0.99 | 1.04 |
| Benzoic acid | 0.58 | 1.00 | 1.00 | 1.19 | 0.70 | 1.90 | 1.31 | 0.78 | 1.18 | 0.68 | 1.98 |  | 0.97 | 0.59 | 2.17 | 0.89 | 0.62 | 0.61 | 1.94 | 0.97 | 2.09 | 1.22 | 1.05 | 1.01 | 1.56 |
| Butyric acid, 4-amino- | 1.12 | 1.03 | 1.01 | 0.76 | 0.99 | 0.94 | 0.87 | 0.93 | 0.85 | 0.83 | 1.02 |  | 1.13 | 1.04 | 0.92 | 1.08 | 1.06 | 1.11 | 1.08 | 0.93 | 0.92 | 1.10 | 1.05 | 0.98 | 1.03 |
| Eicosanoic acid methyl ester, n- | 1.02 | 0.99 | 1.02 | 1.01 | 0.91 | 0.94 | 0.93 | 0.82 | 0.77 | 0.80 | 0.96 |  | 0.91 | 0.93 | 0.94 | 1.03 | 1.01 | 0.94 | 0.91 | 0.90 | 0.91 | 1.03 | 1.00 | 0.83 | 0.87 |
| Fructose, D- | 1.01 | 0.99 | 0.88 | 0.86 | 1.01 | 1.03 | 0.77 | 0.76 | 0.90 | 0.90 | 0.84 |  | 0.84 | 0.58 | 0.57 | 1.19 | 1.18 | 1.23 | 1.21 | 1.09 | 1.13 | 0.71 | 0.72 | 0.79 | 0.81 |
| Fucose, DL- | 1.17 | 1.13 | 0.91 | 0.99 | 0.97 | 1.01 | 0.87 | 0.81 | 1.12 | 1.14 | 0.93 |  | 0.90 | 0.62 | 0.63 | 1.15 | 1.23 | 0.89 | 0.89 | 0.81 | 0.85 | 0.88 | 0.85 | 1.02 | 1.04 |
| Fumaric acid | 1.11 | 1.12 | 1.02 | 0.98 | 0.93 | 0.93 | 1.11 | 1.07 | 0.62 | 0.64 | 1.33 |  | 1.38 | 0.75 | 0.76 | 0.46 | 0.47 | 1.13 | 1.12 | 1.19 | 1.20 | 1.50 | 1.53 | 1.40 | 1.41 |
| Galactinol | 1.34 | 1.19 | 0.97 | 1.03 | 0.67 | 0.87 | 1.16 | 1.05 | 1.48 | 1.54 | 1.13 |  | 1.00 | 0.58 | 0.75 | 1.10 | 1.16 | 2.90 | 3.24 | 0.84 | 1.07 | 3.95 | 3.61 | 6.73 | 7.42 |
| Galactose, D- | 1.17 | 1.19 | 0.89 | 1.11 | 0.70 | 0.74 | 0.80 | 0.77 | 0.71 | 0.84 | 0.83 |  | 0.80 | 0.43 | 0.42 | 2.02 | 2.13 | 2.46 | 2.37 | 0.95 | 1.03 | 0.93 | 0.94 | 1.09 | 1.08 |
| Gluconic acid-1,5-lactone, D- | 1.02 | 1.02 | 0.96 | 0.98 | 1.02 | 0.89 | 0.94 | 0.95 | 0.94 | 0.96 | 0.83 |  | 1.01 | 0.82 | 0.63 | 1.15 | 1.12 | 1.01 | 0.87 | 0.90 | 0.76 | 1.17 | 1.17 | 1.03 | 0.96 |
| Glucopyranoside, 1-O-methyl-, alpha-D- | 1.32 | 1.13 | 0.90 | 1.05 | 0.95 | 0.93 | 0.84 | 0.70 | 1.07 | 1.04 | 0.77 |  | 0.81 | 0.47 | 0.48 | 1.18 | 1.24 | 0.97 | 0.94 | 0.62 | 1.00 | 0.63 | 0.54 | 0.58 | 0.62 |
| Glucose, D- | 1.07 | 1.09 | 1.02 | 0.96 | 0.98 | 0.95 | 0.80 | 0.78 | 0.83 | 0.82 | 0.79 |  | 0.80 | 0.51 | 0.51 | 1.15 | 1.11 | 1.09 | 1.10 | 1.06 | 1.04 | 0.72 | 0.72 | 0.55 | 0.55 |
| Glucosone, 3-deoxy- | 1.02 | 0.98 | 0.85 | 0.86 | 1.03 | 1.06 | 0.73 | 0.70 | 0.91 | 0.90 | 0.79 |  | 0.78 | 0.51 | 0.51 | 1.21 | 1.21 | 1.25 | 1.23 | 1.11 | 1.16 | 0.64 | 0.65 | 0.76 | 0.76 |
| Glutamic acid, DL- | 1.04 | 0.97 | 0.91 | 0.82 | 1.11 | 1.03 | 0.70 | 0.62 | 1.00 | 1.02 | 0.70 |  | 0.64 | 0.64 | 0.67 | 1.17 | 1.19 | 0.73 | 0.71 | 0.59 | 0.63 | 0.78 | 0.81 | 0.48 | 0.50 |
| Glutamine, DL- | 1.06 | 0.92 | 0.90 | 0.94 | 1.33 | 1.44 | 0.36 | 0.44 | 1.11 | 1.28 | 0.42 |  | 0.49 | 0.60 | 0.53 | 1.15 | 1.07 | 0.52 | 0.53 | 0.47 | 0.47 | 0.60 | 0.57 | 0.36 | 0.33 |
| Glyceric acid-3-phosphate, D- | 1.34 | 1.05 | 0.83 | 1.12 | 0.94 | 0.95 | 0.89 | 0.73 | 0.90 | 1.01 | 0.74 |  | 0.74 | 0.47 | 0.47 | 1.30 | 1.54 | 0.91 | 0.90 | 0.98 | 1.08 | 0.70 | 0.65 | 0.52 | 0.50 |
| Glycine | 1.03 | 1.01 | 0.95 | 0.91 | 1.01 | 0.99 | 0.63 | 0.66 | 0.88 | 1.09 | 0.68 |  | 0.71 | 0.37 | 0.33 | 0.78 | 0.77 | 0.66 | 0.66 | 0.70 | 0.55 | 0.61 | 0.60 | 0.35 | 0.34 |
| Hexadecanoic acid methyl ester | 1.01 | 1.01 | 1.06 | 0.99 | 0.92 | 0.89 | 1.01 | 0.95 | 0.80 | 0.83 | 1.03 |  | 1.06 | 1.05 | 0.99 | 1.01 | 0.98 | 1.06 | 1.01 | 1.00 | 0.94 | 1.17 | 1.15 | 0.98 | 1.01 |
| Hexanoic acid, 2-ethyl- | 1.77 | 0.94 | 1.27 | 0.93 | 1.06 | 0.90 | 1.17 | 1.59 | 0.91 | 0.95 | 1.10 |  | 1.54 | 0.99 | 1.15 | 1.32 | 1.30 | 1.25 | 1.16 | 1.20 | 1.06 | 1.33 | 1.46 | 1.19 | 1.13 |
| Idose | 1.07 | 1.09 | 1.02 | 0.96 | 0.98 | 0.95 | 0.80 | 0.78 | 0.83 | 0.82 | 0.79 |  | 0.80 | 0.51 | 0.51 | 1.15 | 1.11 | 1.09 | 1.10 | 1.06 | 1.04 | 0.72 | 0.72 | 0.55 | 0.55 |
| Inositol, myo- | 1.00 | 1.00 | 0.88 | 0.88 | 1.01 | 1.03 | 0.92 | 0.92 | 1.01 | 1.00 | 0.94 |  | 0.95 | 0.68 | 0.68 | 1.17 | 1.18 | 0.98 | 0.97 | 0.84 | 0.86 | 1.04 | 1.03 | 1.11 | 1.12 |
| Isobutyric acid, 2-amino- | 1.11 | 1.08 | 0.84 | 0.71 | 1.01 | 0.99 | 0.75 | 0.57 | 1.23 | 2.62 | 0.69 |  | 0.52 | 0.14 | 0.14 | 0.53 | 0.58 | 0.41 | 0.39 | 0.45 | 0.29 | 0.33 | 0.31 | 0.14 | 0.16 |
| Isoleucine, L- | 1.34 | 1.04 | 1.00 | 1.00 | 0.85 | 0.87 | 0.84 | 0.79 | 1.00 | 1.08 | 0.84 |  | 0.82 | 0.66 | 0.66 | 1.51 | 1.53 | 1.60 | 1.56 | 0.92 | 0.93 | 1.20 | 1.17 | 1.28 | 1.33 |
| Lactic acid, DL- | 1.41 | 0.93 | 1.08 | 1.07 | 0.54 | 0.54 | 0.71 | 0.62 | 0.27 | 0.42 | 0.94 |  | 0.79 | 0.62 | 0.63 | 1.80 | 1.77 | 1.89 | 1.80 | 1.22 | 1.34 | 1.52 | 1.72 | 1.49 | 1.63 |
| Leucine, DL- | 1.09 | 1.02 | 1.00 | 0.60 | 0.94 | 1.00 | 1.57 | 1.11 | 1.04 | 1.40 | 1.73 |  | 1.04 | 0.84 | 1.12 | 1.03 | 1.14 | 1.16 | 1.02 | 0.84 | 0.97 | 1.20 | 1.05 | 0.91 | 1.05 |
| Lysine, L- | 1.29 | 1.02 | 0.91 | 1.03 | 0.91 | 0.98 | 0.67 | 0.58 | 1.00 | 1.14 | 0.71 |  | 0.72 | 0.68 | 0.66 | 2.06 | 2.06 | 1.91 | 1.95 | 1.02 | 1.10 | 1.27 | 1.21 | 1.31 | 1.31 |
| Malic acid, DL- | 1.61 | 1.24 | 1.00 | 1.00 | 0.89 | 0.98 | 1.17 | 1.12 | 1.08 | 1.13 | 1.40 |  | 1.38 | 0.58 | 0.55 | 0.77 | 0.77 | 0.47 | 0.48 | 0.81 | 0.86 | 1.14 | 1.16 | 1.10 | 1.07 |
| Maltose, D- | 1.08 | 1.03 | 0.83 | 0.91 | 0.97 | 1.06 | 0.88 | 0.81 | 1.43 | 1.42 | 0.92 |  | 0.90 | 0.39 | 0.39 | 1.06 | 1.10 | 0.57 | 0.57 | 0.57 | 0.61 | 0.51 | 0.49 | 0.53 | 0.56 |
| Mannose, D- | 1.09 | 1.24 | 1.09 | 0.91 | 0.31 | 0.34 | 1.56 | 0.70 | 0.40 | 0.38 | 0.30 |  | 0.28 | 0.14 | 0.15 | 0.82 | 0.88 | 0.99 | 1.03 | 0.45 | 0.57 | 0.38 | 0.65 | 0.30 | 0.41 |
| Methionine, DL- | 1.16 | 1.00 | 0.82 | 0.86 | 1.00 | 1.04 | 0.59 | 0.55 | 0.89 | 0.95 | 0.61 |  | 0.61 | 0.33 | 0.33 | 1.00 | 1.00 | 0.66 | 0.65 | 0.48 | 0.48 | 0.64 | 0.65 | 0.59 | 0.60 |
| Nicotinic acid | 1.18 | 1.05 | 1.02 | 0.98 | 0.78 | 0.73 | 1.34 | 1.33 | 1.59 | 1.94 | 1.30 |  | 1.18 | 0.95 | 0.94 | 0.95 | 1.00 | 0.76 | 0.74 | 0.82 | 0.90 | 0.97 | 0.92 | 0.77 | 0.76 |
| Octadecanoic acid, n- | 1.08 | 1.06 | 1.17 | 0.94 | 0.94 | 0.83 | 0.85 | 0.88 | 0.80 | 0.90 | 0.91 |  | 0.95 | 0.89 | 0.89 | 1.11 | 0.96 | 0.93 | 1.06 | 0.98 | 0.90 | 1.01 | 1.09 | 0.88 | 0.84 |
| Ornithine, DL- | 1.11 | 0.88 | 0.87 | 0.89 | 1.62 | 1.65 | 0.52 | 0.49 | 1.53 | 1.59 | 0.51 |  | 0.53 | 0.59 | 0.60 | 2.22 | 2.21 | 1.94 | 1.97 | 0.87 | 0.92 | 1.18 | 1.14 | 1.08 | 1.17 |
| Phenylalanine, DL- | 1.17 | 1.00 | 0.86 | 0.96 | 1.00 | 1.08 | 0.56 | 0.50 | 0.80 | 0.94 | 0.61 |  | 0.58 | 0.40 | 0.41 | 1.12 | 1.15 | 0.96 | 0.78 | 0.59 | 0.63 | 0.78 | 0.78 | 0.66 | 0.67 |
| Proline, L- | 1.00 | 1.00 | 0.93 | 0.89 | 1.11 | 1.08 | 0.47 | 0.46 | 0.78 | 0.80 | 0.39 |  | 0.39 | 0.24 | 0.22 | 0.85 | 0.85 | 0.84 | 0.83 | 0.29 | 0.29 | 0.84 | 0.81 | 0.84 | 0.86 |
| Putrescine | 1.03 | 0.97 | 0.82 | 0.88 | 1.32 | 1.41 | 0.59 | 0.60 | 1.85 | 1.68 | 0.80 |  | 0.89 | 0.64 | 0.62 | 1.37 | 1.40 | 0.61 | 0.62 | 0.72 | 0.76 | 0.58 | 0.55 | 0.31 | 0.30 |
| Pyridine, 3-hydroxy- | 1.01 | 0.99 | 0.93 | 0.78 | 1.08 | 1.03 | 1.21 | 1.14 | 1.24 | 1.21 | 1.36 |  | 1.26 | 0.95 | 0.93 | 1.16 | 1.19 | 1.03 | 0.98 | 0.90 | 0.91 | 1.27 | 1.26 | 1.06 | 1.11 |
| Pyruvic acid | 1.26 | 1.15 | 0.99 | 1.01 | 0.66 | 0.73 | 0.91 | 0.94 | 0.95 | 1.03 | 1.17 |  | 1.24 | 0.48 | 0.45 | 1.79 | 1.82 | 1.72 | 1.71 | 0.88 | 0.90 | 1.26 | 1.20 | 1.29 | 1.14 |
| Ribose, D- | 1.01 | 1.01 | 1.00 | 1.00 | 0.87 | 0.87 | 0.95 | 0.95 | 0.89 | 0.88 | 0.98 |  | 0.98 | 0.95 | 0.95 | 0.89 | 0.89 | 0.98 | 0.98 | 0.92 | 0.92 | 1.07 | 1.08 | 0.93 | 0.93 |
| Ribose-5-phosphate, D- | 1.15 | 1.07 | 0.82 | 1.00 | 1.00 | 1.00 | 0.85 | 0.62 | 0.95 | 0.86 | 0.81 |  | 0.81 | 0.43 | 0.47 | 0.97 | 1.00 | 0.62 | 0.62 | 0.65 | 0.76 | 0.87 | 0.84 | 0.75 | 0.73 |
| Salicylic acid | 1.05 | 1.16 | 0.90 | 1.02 | 0.95 | 0.98 | 0.90 | 0.99 | 0.99 | 1.02 | 1.06 |  | 0.90 | 0.96 | 1.05 | 1.06 | 1.19 | 0.84 | 1.05 | 0.95 | 1.04 | 0.97 | 1.03 | 1.04 | 0.86 |
| Serine, DL- | 1.02 | 0.98 | 0.92 | 0.89 | 1.05 | 1.04 | 0.76 | 0.77 | 0.98 | 1.01 | 0.82 |  | 0.83 | 0.78 | 0.77 | 1.50 | 1.48 | 1.22 | 1.22 | 0.76 | 0.76 | 1.11 | 1.10 | 1.38 | 1.41 |
| Shikimic acid | 1.14 | 1.13 | 1.03 | 0.97 | 0.91 | 0.89 | 0.88 | 0.89 | 0.88 | 0.89 | 0.86 |  | 0.89 | 0.61 | 0.60 | 1.03 | 1.05 | 0.79 | 0.79 | 0.89 | 0.91 | 0.90 | 0.91 | 0.72 | 0.73 |
| Spermidine | 1.02 | 0.98 | 0.77 | 0.85 | 1.61 | 1.89 | 0.32 | 0.30 | 1.70 | 1.81 | 0.75 |  | 0.76 | 0.55 | 0.54 | 1.61 | 1.65 | 1.27 | 1.34 | 0.90 | 0.94 | 1.15 | 1.10 | 1.19 | 1.20 |
| Stigmasterol | 1.04 | 0.99 | 1.05 | 1.01 | 0.91 | 0.92 | 0.87 | 0.71 | 0.68 | 0.67 | 0.85 |  | 0.82 | 0.98 | 0.96 | 1.08 | 0.98 | 1.02 | 0.98 | 0.97 | 0.94 | 1.11 | 1.08 | 0.87 | 0.94 |
| Succinic acid | 1.43 | 1.13 | 0.98 | 1.02 | 0.82 | 0.58 | 1.22 | 1.10 | 1.16 | 1.14 | 0.90 |  | 1.56 | 0.65 | 0.49 | 0.95 | 0.95 | 0.77 | 0.79 | 0.89 | 0.73 | 1.05 | 1.03 | 0.95 | 0.73 |
| Sucrose, D- | 1.03 | 1.04 | 1.06 | 0.90 | 0.97 | 0.92 | 0.93 | 1.01 | 0.76 | 0.79 | 1.03 |  | 1.10 | 0.97 | 0.95 | 1.07 | 1.04 | 1.09 | 1.09 | 1.02 | 0.99 | 1.21 | 1.20 | 1.02 | 1.08 |
| Tagatose, D- | 1.06 | 1.02 | 1.07 | 0.98 | 0.98 | 0.91 | 0.97 | 0.99 | 0.87 | 0.91 | 1.07 |  | 1.07 | 1.03 | 0.97 | 1.05 | 1.06 | 1.07 | 1.02 | 0.98 | 0.95 | 1.15 | 1.15 | 0.97 | 1.02 |
| Tetradecanoic acid methyl ester, n- | 1.02 | 1.01 | 1.06 | 0.99 | 0.93 | 0.89 | 0.99 | 0.90 | 0.81 | 0.85 | 1.01 |  | 1.02 | 1.00 | 0.95 | 1.00 | 0.99 | 1.02 | 0.96 | 0.96 | 0.80 | 1.12 | 1.10 | 0.92 | 0.96 |
| Trehalose, alpha,alpha'-, D- | 1.24 | 1.25 | 0.96 | 1.04 | 0.64 | 0.84 | 0.79 | 0.79 | 0.77 | 0.77 | 0.84 |  | 0.71 | 0.35 | 0.42 | 0.91 | 0.97 | 0.45 | 0.48 | 0.70 | 0.82 | 0.72 | 0.69 | 0.45 | 0.56 |
| Triacontanoic acid methyl ester | 1.01 | 1.00 | 1.06 | 1.00 | 0.91 | 0.90 | 0.88 | 0.78 | 0.68 | 0.68 | 0.86 |  | 0.87 | 1.02 | 0.97 | 1.04 | 0.99 | 1.02 | 0.98 | 0.97 | 0.94 | 1.12 | 1.09 | 0.89 | 0.94 |
| Tyrosine, DL- | 1.73 | 0.98 | 1.02 | 1.09 | 0.75 | 0.79 | 0.61 | 0.55 | 0.61 | 0.95 | 0.62 |  | 0.61 | 0.81 | 0.89 | 2.30 | 2.38 | 2.38 | 2.37 | 1.10 | 1.23 | 1.58 | 1.44 | 1.57 | 1.63 |
| Urea | 1.49 | 0.96 | 1.04 | 1.05 | 0.55 | 0.53 | 0.54 | 0.50 | 0.21 | 0.30 | 0.68 |  | 0.67 | 0.48 | 0.51 | 3.27 | 3.19 | 3.32 | 3.30 | 1.24 | 1.30 | 2.02 | 2.00 | 1.94 | 1.97 |
| Valine, DL- | 1.31 | 1.11 | 0.98 | 0.97 | 0.99 | 1.01 | 0.78 | 0.77 | 1.20 | 1.23 | 0.86 |  | 0.86 | 0.54 | 0.53 | 1.27 | 1.27 | 1.23 | 1.22 | 0.72 | 0.73 | 1.04 | 1.01 | 1.16 | 1.18 |
| Xylose, D- | 1.18 | 1.13 | 0.88 | 1.01 | 0.96 | 0.99 | 0.68 | 0.65 | 1.14 | 1.29 | 0.79 |  | 0.77 | 0.48 | 0.51 | 1.09 | 1.08 | 0.57 | 0.59 | 0.69 | 0.69 | 0.63 | 0.65 | 0.59 | 0.64 |
|  |  |  |  |  |  |  |  |  |  |  |  |  |  |  |  |  |  |  |  |  |  |  |  |  |  |
|  | IPGAM2-2-1 | IPGAM2-2-2 | IPGAM2-2-3 | IPGAM2-2-4 | IPGAM2-2-5 | IPGAM2-2-6 | Los2-1 | Los2-2 | Los2-3 | Los2-4 | Los2-5 |  | Los2-6 | PKc3-1 | PKc3-2 | PKc3-3 | PKc3-4 | PKc3-5 | PKc3-6 | PKc4-1 | PKc4-2 | PKc4-3 | PKc4-4 | PKc4-5 | PKc4-6 |
| Aconitic acid, cis- | 0.30 | 0.27 | 0.83 | 0.18 | 0.26 | 0.26 | 0.38 | 0.11 | 0.15 | 0.33 | 0.11 |  | 0.14 | 0.39 | 0.29 | 0.20 | 0.19 | 0.37 | 0.44 | 0.18 | 0.19 | 0.30 | 0.66 | 0.54 | 0.39 |
| Adipic acid | 0.37 | 0.35 | 0.34 | 0.33 | 0.33 | 0.30 | 0.28 | 0.23 | 0.24 | 0.30 | 0.26 |  | 0.27 | 0.20 | 0.24 | 0.24 | 0.34 | 0.27 | 0.24 | 0.22 | 0.22 | 0.20 | 0.25 | 0.28 | 0.21 |
| Alanine, DL- | 0.67 | 0.73 | 1.15 | 1.10 | 1.08 | 1.20 | 0.66 | 0.66 | 0.60 | 1.01 | 1.14 |  | 1.16 | 0.85 | 1.01 | 0.86 | 0.92 | 0.96 | 1.02 | 1.11 | 1.17 | 1.60 | 1.69 | 1.12 | 1.18 |
| Altrose, D- | 0.67 | 0.68 | 0.54 | 0.53 | 0.59 | 0.57 | 0.35 | 0.34 | 0.35 | 0.26 | 0.26 |  | 0.26 | 0.45 | 0.45 | 0.25 | 0.25 | 0.41 | 0.43 | 0.36 | 0.36 | 0.90 | 0.89 | 1.17 | 1.13 |
| Arginine, DL-, -NH3 | 0.54 | 0.50 | 0.35 | 0.40 | 0.25 | 0.23 | 0.07 | 0.06 | 0.07 | 0.07 | 0.07 |  | 0.08 | 0.43 | 0.39 | 0.15 | 0.14 | 1.05 | 1.05 | 0.90 | 0.78 | 0.91 | 0.96 | 0.61 | 0.58 |
| Asparagine, DL- | 0.38 | 0.35 | 0.27 | 0.28 | 0.23 | 0.24 | 0.18 | 0.20 | 0.22 | 0.31 | 0.30 |  | 0.33 | 0.25 | 0.24 | 0.11 | 0.10 | 0.45 | 0.42 | 0.34 | 0.33 | 0.56 | 0.62 | 0.50 | 0.45 |
| Aspartic acid, L- | 0.84 | 0.86 | 0.85 | 0.84 | 0.79 | 0.81 | 0.31 | 0.30 | 0.28 | 0.32 | 0.33 |  | 0.36 | 0.71 | 0.75 | 0.71 | 0.63 | 0.73 | 0.75 | 1.22 | 1.24 | 1.01 | 1.07 | 0.81 | 0.77 |
| Benzoic acid | 1.13 | 0.63 | 0.82 | 0.96 | 1.15 | 1.05 | 0.87 | 0.61 | 0.98 | 0.77 | 2.80 |  | 1.25 | 1.05 | 0.75 | 2.88 | 0.95 | 1.84 | 0.75 | 0.83 | 1.71 | 1.81 | 0.89 | 1.51 | 0.86 |
| Butyric acid, 4-amino- | 0.93 | 0.92 | 0.91 | 0.86 | 1.07 | 0.93 | 0.99 | 0.93 | 0.96 | 0.91 | 0.78 |  | 0.90 | 0.93 | 0.87 | 0.61 | 0.79 | 0.83 | 0.90 | 0.86 | 0.75 | 0.84 | 0.86 | 1.00 | 0.90 |
| Eicosanoic acid methyl ester, n- | 0.91 | 0.89 | 0.90 | 0.86 | 0.95 | 0.90 | 0.80 | 0.75 | 0.78 | 0.83 | 0.87 |  | 0.81 | 0.84 | 0.78 | 0.83 | 0.74 | 0.90 | 0.81 | 0.83 | 0.86 | 0.85 | 0.84 | 0.93 | 0.86 |
| Fructose, D- | 0.91 | 0.91 | 0.66 | 0.67 | 0.70 | 0.68 | 0.26 | 0.26 | 0.27 | 0.17 | 0.18 |  | 0.18 | 0.36 | 0.36 | 0.18 | 0.17 | 0.41 | 0.41 | 0.44 | 0.45 | 0.87 | 0.87 | 1.35 | 1.33 |
| Fucose, DL- | 1.07 | 1.08 | 0.83 | 0.80 | 0.83 | 0.82 | 0.51 | 0.53 | 0.57 | 0.67 | 0.70 |  | 0.72 | 0.81 | 0.82 | 0.76 | 0.74 | 0.84 | 0.83 | 0.95 | 0.97 | 1.06 | 1.09 | 0.89 | 0.87 |
| Fumaric acid | 0.48 | 0.48 | 0.56 | 0.57 | 1.10 | 1.10 | 0.70 | 0.73 | 0.75 | 0.42 | 0.46 |  | 0.48 | 1.04 | 1.05 | 1.08 | 1.03 | 0.29 | 0.30 | 0.33 | 0.35 | 0.93 | 0.95 | 1.10 | 1.07 |
| Galactinol | 1.92 | 1.86 | 1.42 | 1.37 | 1.91 | 1.96 | 1.02 | 1.04 | 1.07 | 1.39 | 1.80 |  | 1.49 | 4.29 | 4.32 | 2.84 | 2.15 | 0.88 | 0.73 | 2.41 | 2.89 | 2.61 | 2.28 | 2.87 | 2.82 |
| Galactose, D- | 0.94 | 1.15 | 0.67 | 0.62 | 0.83 | 0.78 | 0.45 | 0.47 | 0.48 | 0.66 | 0.69 |  | 0.89 | 0.66 | 0.74 | 0.67 | 0.63 | 0.79 | 0.99 | 0.52 | 0.62 | 0.91 | 1.00 | 0.94 | 0.99 |
| Gluconic acid-1,5-lactone, D- | 1.00 | 1.00 | 0.92 | 0.98 | 0.97 | 0.93 | 1.07 | 1.03 | 1.05 | 1.22 | 0.92 |  | 1.20 | 0.96 | 0.96 | 0.70 | 0.91 | 0.85 | 1.00 | 0.97 | 0.79 | 0.97 | 1.07 | 1.06 | 1.04 |
| Glucopyranoside, 1-O-methyl-, alpha-D- | 0.61 | 0.43 | 0.34 | 0.50 | 0.53 | 0.45 | 0.40 | 0.29 | 0.40 | 0.52 | 0.46 |  | 0.54 | 0.49 | 0.48 | 0.34 | 0.21 | 0.52 | 0.38 | 0.45 | 0.47 | 0.76 | 0.74 | 1.09 | 1.05 |
| Glucose, D- | 0.67 | 0.68 | 0.54 | 0.53 | 0.59 | 0.57 | 0.35 | 0.34 | 0.35 | 0.26 | 0.26 |  | 0.26 | 0.45 | 0.45 | 0.25 | 0.25 | 0.41 | 0.43 | 0.36 | 0.36 | 0.90 | 0.89 | 1.17 | 1.13 |
| Glucosone, 3-deoxy- | 0.88 | 0.88 | 0.59 | 0.60 | 0.62 | 0.60 | 0.21 | 0.22 | 0.23 | 0.13 | 0.15 |  | 0.15 | 0.30 | 0.31 | 0.15 | 0.14 | 0.35 | 0.35 | 0.38 | 0.40 | 0.85 | 0.87 | 1.39 | 1.37 |
| Glutamic acid, DL- | 0.59 | 0.65 | 0.70 | 0.66 | 0.46 | 0.54 | 0.33 | 0.29 | 0.28 | 0.41 | 0.40 |  | 0.47 | 0.46 | 0.56 | 0.46 | 0.41 | 0.80 | 0.81 | 0.76 | 0.74 | 0.87 | 0.85 | 0.93 | 0.90 |
| Glutamine, DL- | 0.40 | 0.37 | 0.41 | 0.41 | 0.37 | 0.34 | 0.30 | 0.33 | 0.34 | 0.40 | 0.38 |  | 0.45 | 0.40 | 0.35 | 0.15 | 0.15 | 0.65 | 0.67 | 0.53 | 0.51 | 0.70 | 0.74 | 0.69 | 0.59 |
| Glyceric acid-3-phosphate, D- | 0.64 | 0.63 | 0.50 | 0.46 | 0.49 | 0.49 | 0.38 | 0.38 | 0.40 | 0.69 | 0.69 |  | 0.75 | 0.45 | 0.66 | 0.27 | 0.30 | 0.73 | 0.59 | 0.53 | 0.49 | 0.82 | 1.08 | 0.85 | 0.74 |
| Glycine | 0.31 | 0.31 | 0.41 | 0.42 | 0.41 | 0.41 | 0.11 | 0.10 | 0.20 | 0.13 | 0.12 |  | 0.14 | 0.22 | 0.24 | 0.72 | 0.16 | 0.40 | 0.42 | 0.33 | 0.31 | 0.71 | 0.71 | 0.81 | 0.80 |
| Hexadecanoic acid methyl ester | 1.00 | 1.00 | 1.04 | 1.01 | 1.12 | 1.07 | 0.95 | 0.92 | 0.91 | 0.93 | 0.90 |  | 0.91 | 0.97 | 0.93 | 0.88 | 0.87 | 0.94 | 0.89 | 0.91 | 0.89 | 0.92 | 0.89 | 1.04 | 0.97 |
| Hexanoic acid, 2-ethyl- | 1.04 | 1.07 | 1.08 | 0.88 | 1.07 | 1.15 | 0.81 | 0.76 | 0.89 | 1.04 | 1.19 |  | 1.33 | 1.06 | 1.02 | 0.76 | 0.86 | 0.98 | 1.01 | 1.20 | 1.21 | 1.26 | 1.18 | 1.45 | 1.09 |
| Idose | 0.67 | 0.68 | 0.54 | 0.53 | 0.59 | 0.57 | 0.35 | 0.34 | 0.35 | 0.26 | 0.26 |  | 0.26 | 0.45 | 0.45 | 0.25 | 0.25 | 0.41 | 0.43 | 0.36 | 0.36 | 0.90 | 0.89 | 1.17 | 1.13 |
| Inositol, myo- | 0.92 | 0.92 | 0.78 | 0.79 | 0.92 | 0.90 | 0.61 | 0.61 | 0.62 | 0.70 | 0.70 |  | 0.71 | 0.89 | 0.90 | 0.82 | 0.79 | 0.83 | 0.82 | 0.97 | 0.97 | 1.14 | 1.15 | 1.04 | 1.03 |
| Isobutyric acid, 2-amino- | 0.15 | 0.15 | 0.20 | 0.21 | 0.18 | 0.20 | 0.05 | 0.05 | 0.09 | 0.07 | 0.06 |  | 0.06 | 0.10 | 0.11 | 0.79 | 0.08 | 0.22 | 0.20 | 0.16 | 0.17 | 0.73 | 0.75 | 0.68 | 0.69 |
| Isoleucine, L- | 1.20 | 1.18 | 1.04 | 1.01 | 1.08 | 1.04 | 0.56 | 0.55 | 0.59 | 0.69 | 0.68 |  | 0.73 | 1.05 | 1.06 | 0.69 | 0.73 | 1.42 | 1.43 | 0.86 | 0.85 | 1.24 | 1.29 | 1.09 | 1.02 |
| Lactic acid, DL- | 1.21 | 1.23 | 1.13 | 1.25 | 1.52 | 1.45 | 0.48 | 0.45 | 0.51 | 0.57 | 0.35 |  | 0.43 | 1.25 | 1.38 | 0.86 | 0.81 | 1.47 | 1.50 | 0.55 | 0.52 | 1.25 | 1.17 | 1.39 | 1.31 |
| Leucine, DL- | 1.21 | 1.16 | 1.19 | 0.97 | 1.04 | 1.11 | 0.84 | 0.76 | 0.60 | 0.77 | 1.00 |  | 0.77 | 0.95 | 0.96 | 1.73 | 0.87 | 1.43 | 0.95 | 1.01 | 1.45 | 1.34 | 1.17 | 1.21 | 0.92 |
| Lysine, L- | 1.21 | 1.21 | 1.02 | 0.98 | 1.03 | 1.00 | 0.50 | 0.48 | 0.50 | 0.63 | 0.61 |  | 0.71 | 1.10 | 1.16 | 0.68 | 0.65 | 1.77 | 1.81 | 0.88 | 0.89 | 1.42 | 1.55 | 1.23 | 1.15 |
| Malic acid, DL- | 0.55 | 0.55 | 0.56 | 0.57 | 0.65 | 0.64 | 2.22 | 2.27 | 2.48 | 1.65 | 1.72 |  | 1.85 | 0.66 | 0.66 | 0.76 | 0.71 | 0.27 | 0.27 | 0.51 | 0.52 | 0.64 | 0.70 | 0.74 | 0.81 |
| Maltose, D- | 0.64 | 0.65 | 0.33 | 0.35 | 0.37 | 0.38 | 3.39 | 3.45 | 3.50 | 4.69 | 5.00 |  | 4.97 | 0.39 | 0.41 | 0.37 | 0.33 | 0.42 | 0.38 | 0.39 | 0.40 | 0.91 | 0.94 | 0.70 | 0.71 |
| Mannose, D- | 0.28 | 0.26 | 0.87 | 0.16 | 0.26 | 0.25 | 0.42 | 0.09 | 0.13 | 0.08 | 0.07 |  | 0.07 | 0.40 | 0.28 | 0.19 | 0.19 | 0.38 | 0.49 | 0.18 | 0.18 | 0.27 | 0.65 | 0.58 | 0.40 |
| Methionine, DL- | 0.46 | 0.48 | 0.53 | 0.53 | 0.50 | 0.48 | 0.27 | 0.28 | 0.28 | 0.41 | 0.39 |  | 0.44 | 0.46 | 0.44 | 0.34 | 0.33 | 0.62 | 0.62 | 0.66 | 0.68 | 0.89 | 0.95 | 0.75 | 0.69 |
| Nicotinic acid | 1.11 | 1.23 | 1.10 | 1.06 | 1.10 | 1.15 | 3.33 | 3.59 | 4.30 | 4.49 | 4.54 |  | 4.78 | 1.83 | 1.73 | 1.68 | 1.51 | 1.46 | 1.44 | 1.11 | 1.04 | 0.85 | 0.92 | 0.80 | 0.75 |
| Octadecanoic acid, n- | 0.97 | 0.86 | 0.90 | 0.96 | 0.99 | 1.05 | 0.84 | 0.63 | 0.81 | 0.82 | 0.80 |  | 0.82 | 0.83 | 0.89 | 0.75 | 0.78 | 0.89 | 0.84 | 0.88 | 0.77 | 0.80 | 0.89 | 1.02 | 1.01 |
| Ornithine, DL- | 1.11 | 1.10 | 0.99 | 0.92 | 1.07 | 1.05 | 0.21 | 0.20 | 0.21 | 0.19 | 0.17 |  | 0.20 | 1.06 | 1.18 | 0.50 | 0.47 | 2.50 | 2.49 | 0.93 | 0.92 | 1.53 | 1.60 | 1.11 | 1.08 |
| Phenylalanine, DL- | 0.58 | 0.57 | 0.56 | 0.53 | 0.49 | 0.49 | 0.13 | 0.13 | 0.15 | 0.12 | 0.12 |  | 0.13 | 0.48 | 0.50 | 0.41 | 0.37 | 0.71 | 0.70 | 0.55 | 0.58 | 0.87 | 0.91 | 0.71 | 0.68 |
| Proline, L- | 0.26 | 0.26 | 0.28 | 0.28 | 0.23 | 0.22 | 0.16 | 0.15 | 0.15 | 0.18 | 0.19 |  | 0.19 | 0.19 | 0.19 | 0.18 | 0.17 | 0.28 | 0.28 | 0.36 | 0.37 | 0.87 | 0.88 | 1.06 | 0.99 |
| Putrescine | 0.97 | 0.93 | 0.62 | 0.64 | 0.57 | 0.56 | 1.38 | 1.34 | 1.30 | 2.08 | 1.80 |  | 2.20 | 0.68 | 0.66 | 0.48 | 0.51 | 0.88 | 0.92 | 0.61 | 0.55 | 0.71 | 0.73 | 0.72 | 0.68 |
| Pyridine, 3-hydroxy- | 1.07 | 1.10 | 1.07 | 1.11 | 1.04 | 1.03 | 1.05 | 1.12 | 1.28 | 1.27 | 1.28 |  | 1.28 | 1.06 | 1.01 | 1.16 | 1.21 | 1.08 | 1.05 | 1.05 | 1.08 | 1.18 | 1.16 | 1.15 | 1.16 |
| Pyruvic acid | 0.65 | 0.65 | 0.78 | 0.79 | 0.95 | 0.89 | 0.77 | 0.78 | 0.83 | 1.08 | 1.00 |  | 1.13 | 1.04 | 1.06 | 0.58 | 0.58 | 1.13 | 1.28 | 0.62 | 0.63 | 0.99 | 1.08 | 0.88 | 0.84 |
| Ribose, D- | 0.97 | 0.97 | 1.01 | 1.01 | 1.07 | 1.08 | 0.95 | 0.95 | 0.95 | 1.02 | 1.02 |  | 1.03 | 0.96 | 0.96 | 0.89 | 0.89 | 0.92 | 0.92 | 0.93 | 0.93 | 0.88 | 0.88 | 1.04 | 1.04 |
| Ribose-5-phosphate, D- | 0.62 | 0.67 | 0.54 | 0.56 | 0.48 | 0.52 | 0.78 | 0.71 | 0.79 | 1.13 | 1.07 |  | 1.30 | 0.57 | 0.66 | 0.67 | 0.64 | 0.65 | 0.66 | 0.60 | 0.60 | 0.90 | 0.96 | 0.78 | 0.72 |
| Salicylic acid | 1.01 | 0.96 | 1.18 | 1.09 | 1.03 | 1.28 | 1.11 | 1.14 | 1.14 | 1.15 | 1.09 |  | 1.18 | 0.83 | 0.89 | 1.07 | 1.14 | 1.07 | 1.16 | 0.94 | 1.20 | 1.01 | 1.40 | 1.44 | 1.23 |
| Serine, DL- | 1.47 | 1.48 | 1.12 | 1.12 | 1.12 | 1.10 | 0.53 | 0.52 | 0.52 | 0.66 | 0.65 |  | 0.66 | 0.98 | 0.99 | 0.81 | 0.77 | 1.46 | 1.43 | 1.27 | 1.23 | 1.25 | 1.23 | 1.07 | 1.04 |
| Shikimic acid | 0.74 | 0.74 | 0.70 | 0.71 | 0.70 | 0.68 | 0.51 | 0.51 | 0.55 | 0.56 | 0.59 |  | 0.60 | 0.55 | 0.55 | 0.49 | 0.48 | 0.71 | 0.70 | 0.62 | 0.63 | 0.87 | 0.88 | 0.89 | 0.87 |
| Spermidine | 1.45 | 1.44 | 0.90 | 0.93 | 0.86 | 0.85 | 0.67 | 0.63 | 0.66 | 1.15 | 1.17 |  | 1.21 | 0.94 | 0.93 | 0.95 | 0.83 | 1.54 | 1.54 | 1.47 | 1.53 | 1.99 | 2.08 | 1.20 | 1.13 |
| Stigmasterol | 0.92 | 0.92 | 0.94 | 0.86 | 0.99 | 0.93 | 0.81 | 0.80 | 0.79 | 0.85 | 0.83 |  | 0.85 | 0.81 | 0.76 | 0.74 | 0.71 | 0.87 | 0.74 | 0.79 | 0.80 | 0.88 | 0.82 | 0.91 | 0.78 |
| Succinic acid | 0.41 | 0.43 | 0.47 | 0.51 | 0.51 | 0.50 | 0.61 | 0.59 | 0.65 | 0.80 | 0.51 |  | 0.87 | 0.53 | 0.74 | 0.44 | 0.87 | 0.59 | 0.78 | 0.65 | 0.45 | 0.79 | 1.00 | 1.05 | 1.60 |
| Sucrose, D- | 1.04 | 1.04 | 1.08 | 1.06 | 1.17 | 1.14 | 1.05 | 0.98 | 0.98 | 1.03 | 1.00 |  | 0.99 | 1.01 | 0.98 | 0.92 | 0.89 | 0.95 | 0.92 | 0.97 | 0.92 | 0.95 | 0.89 | 1.05 | 1.00 |
| Tagatose, D- | 1.01 | 1.02 | 1.04 | 1.03 | 1.13 | 1.10 | 0.98 | 0.91 | 0.93 | 0.96 | 0.94 |  | 0.93 | 0.96 | 0.93 | 0.88 | 0.83 | 0.95 | 0.92 | 0.94 | 0.91 | 0.95 | 0.93 | 1.08 | 1.01 |
| Tetradecanoic acid methyl ester, n- | 0.98 | 0.98 | 1.02 | 0.97 | 1.09 | 1.03 | 0.94 | 0.90 | 0.91 | 0.94 | 0.91 |  | 0.92 | 0.94 | 0.90 | 0.88 | 0.82 | 0.91 | 0.88 | 0.90 | 0.87 | 0.90 | 0.89 | 1.02 | 0.93 |
| Trehalose, alpha,alpha'-, D- | 0.48 | 0.49 | 0.39 | 0.39 | 0.39 | 0.41 | 0.67 | 0.63 | 0.70 | 0.85 | 1.01 |  | 0.94 | 0.45 | 0.48 | 0.49 | 0.37 | 0.58 | 0.46 | 0.32 | 0.40 | 0.67 | 0.62 | 0.63 | 0.61 |
| Triacontanoic acid methyl ester | 0.94 | 0.93 | 0.95 | 0.89 | 1.01 | 0.98 | 0.86 | 0.83 | 0.81 | 0.87 | 0.83 |  | 0.85 | 0.84 | 0.80 | 0.76 | 0.74 | 0.87 | 0.78 | 0.80 | 0.80 | 0.85 | 0.79 | 0.91 | 0.81 |
| Tyrosine, DL- | 1.51 | 1.53 | 1.38 | 1.16 | 1.30 | 1.23 | 0.48 | 0.47 | 0.54 | 0.50 | 0.51 |  | 0.54 | 1.20 | 1.27 | 0.73 | 0.66 | 2.10 | 2.05 | 0.64 | 0.63 | 1.53 | 1.71 | 1.73 | 1.57 |
| Urea | 1.17 | 1.20 | 1.09 | 1.01 | 1.46 | 1.39 | 0.35 | 0.34 | 0.38 | 0.43 | 0.35 |  | 0.38 | 1.27 | 1.36 | 0.76 | 0.75 | 2.37 | 2.40 | 0.52 | 0.46 | 1.49 | 1.69 | 1.69 | 1.54 |
| Valine, DL- | 0.90 | 0.88 | 0.89 | 0.90 | 0.93 | 0.87 | 0.49 | 0.47 | 0.48 | 0.69 | 0.68 |  | 0.73 | 0.80 | 0.81 | 0.54 | 0.61 | 0.99 | 1.02 | 0.85 | 0.84 | 1.09 | 1.16 | 0.95 | 0.91 |
| Xylose, D- | 0.87 | 0.88 | 0.68 | 0.64 | 0.61 | 0.62 | 0.40 | 0.43 | 0.48 | 0.64 | 0.65 |  | 0.69 | 0.62 | 0.57 | 0.47 | 0.44 | 0.75 | 0.73 | 0.75 | 0.85 | 0.96 | 1.06 | 0.74 | 0.76 |

**Supplementary table5 metabolites normalized by WT of PGAM mutants and overexpressers**

|  | WT1 | WT2 | WT3 | WT4 | WT5 | WT6 | IPGAM1-OE1-1 | IPGAM1-OE1-2 | IPGAM1-OE1-3 | IPGAM1-OE1-4 | IPGAM1-OE1-5 | IPGAM1-OE1-6 | IPGAM1-OE2-1 | IPGAM1-OE2-2 | IPGAM1-OE2-3 | IPGAM1-OE2-4 | IPGAM1-OE2-5 | IPGAM1-OE2-6 | IPGAM1-OE3-1 | IPGAM1-OE3-2 | IPGAM1-OE3-3 | IPGAM1-OE3-4 | IPGAM1-OE3-5 | IPGAM1-OE3-6 |
| --- | --- | --- | --- | --- | --- | --- | --- | --- | --- | --- | --- | --- | --- | --- | --- | --- | --- | --- | --- | --- | --- | --- | --- | --- |
| Aconitic acid, cis- | 1.85 | 1.37 | 1.09 | 0.91 | 0.31 | 0.34 | 4.67 | 4.84 | 4.66 | 4.44 | 4.61 | 5.17 | 0.53 | 0.37 | 0.44 | 0.40 | 0.37 | 0.51 | 1.53 | 1.51 | 1.38 | 1.49 | 1.40 | 1.50 |
| Adipic acid | 1.79 | 1.34 | 1.12 | 0.88 | 0.81 | 0.75 | 0.35 | 0.30 | 0.30 | 0.30 | 0.27 | 0.30 | 0.31 | 0.27 | 0.24 | 0.38 | 0.24 | 0.25 | 0.26 | 0.23 | 0.23 | 0.31 | 0.29 | 0.26 |
| Alanine, DL- | 1.38 | 1.30 | 1.08 | 0.82 | 0.92 | 0.90 | 3.27 | 2.95 | 3.57 | 3.11 | 2.98 | 3.45 | 2.17 | 2.30 | 2.12 | 2.09 | 2.52 | 2.70 | 2.36 | 2.06 | 2.24 | 2.15 | 2.26 | 2.51 |
| Altrose, D- | 1.07 | 1.09 | 1.02 | 0.96 | 0.98 | 0.95 | 0.54 | 0.54 | 0.54 | 0.55 | 0.56 | 0.56 | 0.50 | 0.51 | 0.50 | 0.50 | 0.51 | 0.50 | 0.37 | 0.37 | 0.37 | 0.38 | 0.37 | 0.37 |
| Arginine, DL-, -NH3 | 1.03 | 0.86 | 0.67 | 0.97 | 2.12 | 2.35 | 0.33 | 0.26 | 0.25 | 0.26 | 0.26 | 0.29 | 0.20 | 0.17 | 0.16 | 0.19 | 0.15 | 0.14 | 0.12 | 0.16 | 0.16 | 0.12 | 0.16 | 0.14 |
| Asparagine, DL- | 1.05 | 0.92 | 0.73 | 0.95 | 1.51 | 1.62 | 0.21 | 0.17 | 0.16 | 0.17 | 0.18 | 0.15 | 0.13 | 0.11 | 0.11 | 0.11 | 0.12 | 0.10 | 0.11 | 0.12 | 0.12 | 0.10 | 0.12 | 0.11 |
| Aspartic acid, L- | 1.20 | 0.97 | 0.82 | 0.81 | 1.03 | 1.12 | 0.88 | 0.74 | 0.76 | 0.74 | 0.75 | 0.76 | 0.69 | 0.61 | 0.59 | 0.64 | 0.66 | 0.62 | 0.56 | 0.61 | 0.59 | 0.54 | 0.63 | 0.62 |
| Benzoic acid | 0.58 | 1.00 | 1.00 | 1.19 | 0.70 | 1.90 | 0.96 | 0.87 | 1.54 | 0.86 | 0.90 | 1.05 | 0.58 | 0.67 | 1.97 | 0.50 | 0.52 | 0.56 | 0.79 | 0.75 | 0.82 | 1.04 | 0.65 | 0.80 |
| Butyric acid, 4-amino- | 1.12 | 1.03 | 1.01 | 0.76 | 0.99 | 0.94 | 1.12 | 1.00 | 0.81 | 1.02 | 0.99 | 0.96 | 0.87 | 0.80 | 0.72 | 0.92 | 0.78 | 0.74 | 0.87 | 0.92 | 0.89 | 0.90 | 0.92 | 0.91 |
| Eicosanoic acid methyl ester, n- | 1.02 | 0.99 | 1.02 | 1.01 | 0.91 | 0.94 | 1.03 | 0.90 | 1.01 | 0.92 | 0.88 | 0.86 | 0.84 | 0.81 | 0.92 | 0.77 | 0.78 | 0.77 | 0.92 | 0.92 | 0.90 | 1.00 | 0.94 | 0.95 |
| Fructose, D- | 1.01 | 0.99 | 0.88 | 0.86 | 1.01 | 1.03 | 0.63 | 0.60 | 0.63 | 0.60 | 0.61 | 0.63 | 0.44 | 0.44 | 0.47 | 0.46 | 0.46 | 0.46 | 0.29 | 0.29 | 0.29 | 0.28 | 0.29 | 0.30 |
| Fucose, DL- | 1.17 | 1.13 | 0.91 | 0.99 | 0.97 | 1.01 | 0.65 | 0.59 | 0.63 | 0.60 | 0.61 | 0.61 | 0.49 | 0.48 | 0.49 | 0.50 | 0.51 | 0.51 | 0.57 | 0.55 | 0.57 | 0.51 | 0.55 | 0.57 |
| Fumaric acid | 1.11 | 1.12 | 1.02 | 0.98 | 0.93 | 0.93 | 1.05 | 1.04 | 1.08 | 1.03 | 1.01 | 1.03 | 1.20 | 1.09 | 1.11 | 1.15 | 1.10 | 1.10 | 1.56 | 1.63 | 1.61 | 1.61 | 1.71 | 1.73 |
| Galactinol | 1.34 | 1.19 | 0.97 | 1.03 | 0.67 | 0.87 | 0.40 | 0.32 | 0.50 | 0.36 | 0.34 | 0.38 | 0.21 | 0.21 | 0.30 | 0.23 | 0.23 | 0.23 | 0.28 | 0.28 | 0.29 | 0.28 | 0.28 | 0.30 |
| Galactose, D- | 1.17 | 1.19 | 0.89 | 1.11 | 0.70 | 0.74 | 0.89 | 0.80 | 0.78 | 0.76 | 0.80 | 0.80 | 0.48 | 0.49 | 0.50 | 0.91 | 0.58 | 0.52 | 1.26 | 1.21 | 1.18 | 0.56 | 0.59 | 0.60 |
| Gluconic acid-1,5-lactone, D- | 1.02 | 1.02 | 0.96 | 0.98 | 1.02 | 0.89 | 1.14 | 1.16 | 0.74 | 1.05 | 1.06 | 1.11 | 1.01 | 0.98 | 0.67 | 1.02 | 0.94 | 0.97 | 1.12 | 1.17 | 1.18 | 1.17 | 1.21 | 1.25 |
| Glucopyranoside, 1-O-methyl-, alpha-D- | 1.32 | 1.13 | 0.90 | 1.05 | 0.95 | 0.93 | 0.54 | 0.38 | 0.30 | 0.49 | 0.50 | 0.55 | 0.28 | 0.30 | 0.24 | 0.47 | 0.45 | 0.44 | 0.31 | 0.39 | 0.50 | 0.41 | 0.45 | 0.44 |
| Glucose, D- | 1.07 | 1.09 | 1.02 | 0.96 | 0.98 | 0.95 | 0.54 | 0.54 | 0.54 | 0.55 | 0.56 | 0.56 | 0.50 | 0.51 | 0.50 | 0.50 | 0.51 | 0.50 | 0.37 | 0.37 | 0.37 | 0.38 | 0.37 | 0.37 |
| Glucosone, 3-deoxy- | 1.02 | 0.98 | 0.85 | 0.86 | 1.03 | 1.06 | 0.55 | 0.52 | 0.55 | 0.52 | 0.53 | 0.55 | 0.38 | 0.38 | 0.40 | 0.40 | 0.40 | 0.40 | 0.24 | 0.24 | 0.24 | 0.24 | 0.24 | 0.25 |
| Glutamic acid, DL- | 1.04 | 0.97 | 0.91 | 0.82 | 1.11 | 1.03 | 0.65 | 0.56 | 0.64 | 0.56 | 0.55 | 0.61 | 0.52 | 0.46 | 0.43 | 0.46 | 0.48 | 0.47 | 0.45 | 0.42 | 0.47 | 0.42 | 0.48 | 0.50 |
| Glutamine, DL- | 1.06 | 0.92 | 0.90 | 0.94 | 1.33 | 1.44 | 0.43 | 0.36 | 0.26 | 0.34 | 0.38 | 0.31 | 0.24 | 0.19 | 0.20 | 0.27 | 0.20 | 0.17 | 0.16 | 0.24 | 0.20 | 0.18 | 0.22 | 0.19 |
| Glyceric acid-3-phosphate, D- | 1.34 | 1.05 | 0.83 | 1.12 | 0.94 | 0.95 | 0.58 | 0.52 | 0.36 | 0.46 | 0.49 | 0.46 | 0.33 | 0.31 | 0.36 | 0.36 | 0.41 | 0.40 | 0.46 | 0.47 | 0.46 | 0.43 | 0.50 | 0.53 |
| Glycine | 1.03 | 1.01 | 0.95 | 0.91 | 1.01 | 0.99 | 0.73 | 0.75 | 0.72 | 0.70 | 0.70 | 0.78 | 0.80 | 0.74 | 0.71 | 0.77 | 0.71 | 1.29 | 0.64 | 0.66 | 0.64 | 0.65 | 0.67 | 1.43 |
| Hexadecanoic acid methyl ester | 1.01 | 1.01 | 1.06 | 0.99 | 0.92 | 0.89 | 1.18 | 1.06 | 1.06 | 1.11 | 1.04 | 1.00 | 1.06 | 1.01 | 0.98 | 0.97 | 0.93 | 0.93 | 1.09 | 1.09 | 1.06 | 1.17 | 1.12 | 1.16 |
| Hexanoic acid, 2-ethyl- | 1.77 | 0.94 | 1.27 | 0.93 | 1.06 | 0.90 | 1.43 | 1.61 | 1.01 | 1.10 | 1.52 | 1.08 | 0.94 | 0.96 | 0.91 | 1.17 | 0.90 | 1.00 | 1.05 | 1.15 | 1.17 | 1.16 | 1.27 | 1.37 |
| Idose | 1.07 | 1.09 | 1.02 | 0.96 | 0.98 | 0.95 | 0.54 | 0.54 | 0.54 | 0.55 | 0.56 | 0.56 | 0.50 | 0.51 | 0.50 | 0.50 | 0.51 | 0.50 | 0.37 | 0.37 | 0.37 | 0.38 | 0.37 | 0.37 |
| Inositol, myo- | 1.00 | 1.00 | 0.88 | 0.88 | 1.01 | 1.03 | 0.68 | 0.66 | 0.67 | 0.66 | 0.65 | 0.67 | 0.46 | 0.46 | 0.47 | 0.47 | 0.49 | 0.49 | 0.75 | 0.75 | 0.74 | 0.74 | 0.76 | 0.78 |
| Isobutyric acid, 2-amino- | 1.11 | 1.08 | 0.84 | 0.71 | 1.01 | 0.99 | 0.52 | 0.48 | 0.63 | 0.51 | 0.49 | 0.61 | 0.60 | 0.66 | 0.62 | 0.43 | 0.58 | 4.43 | 0.47 | 0.39 | 0.40 | 0.42 | 0.39 | 2.78 |
| Isoleucine, L- | 1.34 | 1.04 | 1.00 | 1.00 | 0.85 | 0.87 | 1.49 | 1.32 | 1.34 | 1.38 | 1.34 | 1.33 | 1.40 | 1.34 | 1.35 | 1.39 | 1.34 | 1.33 | 1.06 | 1.07 | 1.02 | 1.02 | 1.07 | 1.07 |
| Lactic acid, DL- | 1.41 | 0.93 | 1.08 | 1.07 | 0.54 | 0.54 | 2.36 | 2.47 | 2.34 | 2.30 | 2.32 | 2.36 | 2.35 | 2.29 | 2.29 | 2.35 | 2.17 | 2.17 | 1.70 | 1.78 | 1.68 | 1.85 | 1.79 | 1.85 |
| Leucine, DL- | 1.09 | 1.02 | 1.00 | 0.60 | 0.94 | 1.00 | 1.19 | 1.00 | 1.80 | 1.25 | 0.98 | 0.99 | 0.99 | 1.15 | 1.43 | 0.71 | 0.88 | 1.26 | 1.18 | 1.00 | 1.02 | 1.36 | 1.00 | 1.30 |
| Lysine, L- | 1.29 | 1.02 | 0.91 | 1.03 | 0.91 | 0.98 | 1.30 | 1.13 | 1.11 | 1.16 | 1.17 | 1.17 | 1.25 | 1.21 | 1.16 | 1.30 | 1.25 | 1.25 | 0.76 | 0.79 | 0.78 | 0.74 | 0.83 | 0.81 |
| Malic acid, DL- | 1.61 | 1.24 | 1.00 | 1.00 | 0.89 | 0.98 | 1.22 | 1.02 | 0.99 | 1.00 | 1.03 | 1.02 | 0.98 | 0.82 | 0.81 | 0.93 | 0.95 | 0.90 | 2.04 | 2.31 | 2.25 | 1.96 | 2.35 | 2.29 |
| Maltose, D- | 1.08 | 1.03 | 0.83 | 0.91 | 0.97 | 1.06 | 0.56 | 0.50 | 0.55 | 0.50 | 0.52 | 0.52 | 0.83 | 0.83 | 0.96 | 0.83 | 0.88 | 0.87 | 1.06 | 1.07 | 1.07 | 1.05 | 1.04 | 1.04 |
| Mannose, D- | 1.09 | 1.24 | 1.09 | 0.91 | 0.31 | 0.34 | 4.18 | 4.24 | 4.22 | 4.06 | 4.12 | 4.66 | 0.58 | 0.41 | 0.46 | 0.42 | 0.39 | 0.55 | 1.50 | 1.42 | 1.33 | 1.46 | 1.37 | 1.40 |
| Methionine, DL- | 1.16 | 1.00 | 0.82 | 0.86 | 1.00 | 1.04 | 0.65 | 0.58 | 0.58 | 0.59 | 0.60 | 0.60 | 0.48 | 0.46 | 0.47 | 0.47 | 0.48 | 0.48 | 0.57 | 0.58 | 0.58 | 0.55 | 0.58 | 0.60 |
| Nicotinic acid | 1.18 | 1.05 | 1.02 | 0.98 | 0.78 | 0.73 | 0.92 | 0.72 | 0.67 | 0.62 | 0.70 | 0.69 | 0.83 | 0.78 | 0.77 | 0.75 | 0.73 | 0.78 | 0.88 | 0.87 | 0.85 | 0.88 | 0.85 | 0.96 |
| Octadecanoic acid, n- | 1.08 | 1.06 | 1.17 | 0.94 | 0.94 | 0.83 | 1.16 | 1.07 | 1.01 | 0.88 | 0.83 | 0.84 | 0.84 | 0.93 | 0.84 | 0.75 | 0.83 | 0.89 | 1.21 | 1.18 | 1.05 | 1.28 | 1.19 | 1.15 |
| Ornithine, DL- | 1.11 | 0.88 | 0.87 | 0.89 | 1.62 | 1.65 | 1.79 | 1.55 | 1.54 | 1.57 | 1.57 | 1.61 | 1.57 | 1.51 | 1.44 | 1.50 | 1.55 | 1.49 | 0.92 | 0.97 | 0.90 | 0.91 | 1.00 | 0.95 |
| Phenylalanine, DL- | 1.17 | 1.00 | 0.86 | 0.96 | 1.00 | 1.08 | 0.91 | 0.80 | 0.89 | 0.85 | 0.82 | 0.82 | 0.65 | 0.63 | 0.72 | 0.66 | 0.66 | 0.65 | 0.50 | 0.52 | 0.49 | 0.49 | 0.53 | 0.54 |
| Proline, L- | 1.00 | 1.00 | 0.93 | 0.89 | 1.11 | 1.08 | 0.74 | 0.67 | 0.66 | 0.67 | 0.66 | 0.67 | 0.20 | 0.19 | 0.19 | 0.20 | 0.19 | 0.21 | 0.22 | 0.22 | 0.22 | 0.21 | 0.22 | 0.23 |
| Putrescine | 1.03 | 0.97 | 0.82 | 0.88 | 1.32 | 1.41 | 0.63 | 0.56 | 0.51 | 0.59 | 0.56 | 0.61 | 0.55 | 0.54 | 0.50 | 0.56 | 0.54 | 0.48 | 0.50 | 0.53 | 0.51 | 0.44 | 0.52 | 0.49 |
| Pyridine, 3-hydroxy- | 1.01 | 0.99 | 0.93 | 0.78 | 1.08 | 1.03 | 1.22 | 1.19 | 1.38 | 1.34 | 1.21 | 1.26 | 1.25 | 1.19 | 1.13 | 1.12 | 1.08 | 1.15 | 1.56 | 1.37 | 1.37 | 1.42 | 1.40 | 1.46 |
| Pyruvic acid | 1.26 | 1.15 | 0.99 | 1.01 | 0.66 | 0.73 | 2.91 | 2.72 | 2.49 | 2.66 | 2.60 | 2.66 | 2.09 | 2.08 | 2.08 | 2.06 | 1.99 | 2.00 | 1.49 | 1.58 | 1.59 | 1.48 | 1.62 | 1.65 |
| Ribose, D- | 1.01 | 1.01 | 1.00 | 1.00 | 0.87 | 0.87 | 1.03 | 1.03 | 1.04 | 1.04 | 1.03 | 1.03 | 0.95 | 0.94 | 0.95 | 0.94 | 0.95 | 0.94 | 1.00 | 1.01 | 1.00 | 1.00 | 1.00 | 1.00 |
| Ribose-5-phosphate, D- | 1.15 | 1.07 | 0.82 | 1.00 | 1.00 | 1.00 | 0.74 | 0.66 | 0.66 | 0.69 | 0.72 | 0.88 | 0.58 | 0.60 | 0.50 | 0.58 | 0.66 | 0.67 | 0.90 | 0.90 | 0.92 | 0.88 | 0.94 | 1.03 |
| Salicylic acid | 1.05 | 1.16 | 0.90 | 1.02 | 0.95 | 0.98 | 1.38 | 1.37 | 1.43 | 1.13 | 1.14 | 1.16 | 0.97 | 0.91 | 1.14 | 0.90 | 1.11 | 1.19 | 1.23 | 1.46 | 1.27 | 1.03 | 1.30 | 1.50 |
| Serine, DL- | 1.02 | 0.98 | 0.92 | 0.89 | 1.05 | 1.04 | 1.08 | 1.06 | 1.05 | 1.04 | 1.03 | 1.07 | 0.96 | 0.93 | 0.91 | 0.95 | 0.90 | 0.92 | 0.89 | 0.89 | 0.88 | 0.91 | 0.91 | 0.93 |
| Shikimic acid | 1.14 | 1.13 | 1.03 | 0.97 | 0.91 | 0.89 | 1.02 | 0.98 | 0.99 | 0.96 | 0.97 | 0.98 | 0.74 | 0.75 | 0.76 | 0.76 | 0.76 | 0.78 | 0.87 | 0.90 | 0.89 | 0.87 | 0.88 | 0.92 |
| Spermidine | 1.02 | 0.98 | 0.77 | 0.85 | 1.61 | 1.89 | 1.57 | 1.29 | 1.36 | 1.32 | 1.30 | 1.36 | 0.99 | 0.96 | 1.03 | 1.03 | 0.99 | 0.97 | 0.46 | 0.48 | 0.46 | 0.42 | 0.44 | 0.43 |
| Stigmasterol | 1.04 | 0.99 | 1.05 | 1.01 | 0.91 | 0.92 | 0.95 | 0.76 | 0.78 | 0.82 | 0.73 | 0.70 | 0.68 | 0.63 | 0.70 | 0.71 | 0.68 | 0.69 | 0.80 | 0.79 | 0.72 | 0.84 | 0.77 | 0.78 |
| Succinic acid | 1.43 | 1.13 | 0.98 | 1.02 | 0.82 | 0.58 | 1.34 | 1.18 | 0.69 | 1.29 | 1.31 | 1.38 | 1.22 | 1.18 | 0.71 | 1.43 | 1.36 | 1.47 | 1.87 | 2.11 | 1.94 | 2.25 | 2.09 | 2.47 |
| Sucrose, D- | 1.03 | 1.04 | 1.06 | 0.90 | 0.97 | 0.92 | 1.17 | 1.07 | 1.07 | 1.07 | 1.04 | 1.04 | 1.01 | 0.97 | 0.99 | 1.04 | 0.91 | 0.91 | 1.07 | 1.04 | 1.01 | 1.12 | 1.06 | 1.07 |
| Tagatose, D- | 1.06 | 1.02 | 1.07 | 0.98 | 0.98 | 0.91 | 1.03 | 1.08 | 1.00 | 1.01 | 0.98 | 0.94 | 1.06 | 1.02 | 1.00 | 0.98 | 0.95 | 0.94 | 1.10 | 1.13 | 1.11 | 1.22 | 1.15 | 1.19 |
| Tetradecanoic acid methyl ester, n- | 1.02 | 1.01 | 1.06 | 0.99 | 0.93 | 0.89 | 1.16 | 1.06 | 1.04 | 1.07 | 1.02 | 0.99 | 1.02 | 0.97 | 0.96 | 0.94 | 0.91 | 0.89 | 1.05 | 1.08 | 1.05 | 1.15 | 1.10 | 1.13 |
| Trehalose, alpha,alpha'-, D- | 1.24 | 1.25 | 0.96 | 1.04 | 0.64 | 0.84 | 0.78 | 0.70 | 0.98 | 0.73 | 0.75 | 0.75 | 0.45 | 0.44 | 0.63 | 0.50 | 0.48 | 0.51 | 1.01 | 1.02 | 1.01 | 0.98 | 0.98 | 1.02 |
| Triacontanoic acid methyl ester | 1.01 | 1.00 | 1.06 | 1.00 | 0.91 | 0.90 | 0.97 | 0.83 | 0.82 | 0.87 | 0.79 | 0.75 | 0.74 | 0.70 | 0.75 | 0.75 | 0.71 | 0.71 | 0.82 | 0.81 | 0.77 | 0.89 | 0.81 | 0.83 |
| Tyrosine, DL- | 1.73 | 0.98 | 1.02 | 1.09 | 0.75 | 0.79 | 1.51 | 1.30 | 1.35 | 1.32 | 1.27 | 1.26 | 1.70 | 1.63 | 1.71 | 1.51 | 1.57 | 1.53 | 0.80 | 0.85 | 0.79 | 0.77 | 0.90 | 0.88 |
| Urea | 1.49 | 0.96 | 1.04 | 1.05 | 0.55 | 0.53 | 4.20 | 4.24 | 4.12 | 4.19 | 4.21 | 4.18 | 4.05 | 4.02 | 4.00 | 4.03 | 3.75 | 3.84 | 2.32 | 2.34 | 2.29 | 2.29 | 2.33 | 2.50 |
| Valine, DL- | 1.31 | 1.11 | 0.98 | 0.97 | 0.99 | 1.01 | 1.17 | 1.06 | 1.01 | 1.10 | 1.06 | 1.05 | 0.91 | 0.88 | 0.88 | 0.93 | 0.89 | 0.89 | 0.83 | 0.84 | 0.81 | 0.77 | 0.82 | 0.85 |
| Xylose, D- | 1.18 | 1.13 | 0.88 | 1.01 | 0.96 | 0.99 | 0.77 | 0.72 | 0.76 | 0.68 | 0.72 | 0.70 | 0.54 | 0.51 | 0.52 | 0.48 | 0.57 | 0.53 | 0.60 | 0.65 | 0.67 | 0.61 | 0.68 | 0.64 |

**Supplementary table6 metabolites normalized by WT of PGAM mutants complementation lines**

|  | WT1 | WT2 | WT3 | WT4 | *E.pgam-pgam1/2 -1* | *E.pgam-pgam1/2 -2* | *E.pgam-pgam1/2 -3* | *E.pgam-pgam1/2 -4* | *sdmA-E.pgam-pgam1/2 -1-1* | *sdmA-E.pgam-pgam1/2 -1-2* | *sdmA-E.pgam-pgam1/2 -1-3* | *sdmA-E.pgam-pgam1/2 -1-4* | *sdmA-E.pgam-pgam1/2 -2-1* | *sdmA-E.pgam-pgam1/2 -2-2* | *sdmA-E.pgam-pgam1/2 -2-3* | *sdmA-E.pgam-pgam1/2 -2-4* |
| --- | --- | --- | --- | --- | --- | --- | --- | --- | --- | --- | --- | --- | --- | --- | --- | --- |
| Ribitol | 0.99 | 1.00 | 1.01 | 1.00 | 0.91 | 1.12 | 1.19 | 0.96 | 0.69 | 1.01 | 1.07 | 1.02 | 1.21 | 0.90 | 0.68 | 1.00 |
| Aconitic acid, cis- | 0.97 | 1.01 | 1.00 | 1.03 | 0.81 | 0.37 | 0.52 | 0.39 | 0.31 | 0.20 | 1.01 | 0.46 | 0.59 | 0.17 | 0.31 | 0.52 |
| Alanine | 0.83 | 0.58 | 1.33 | 1.27 | 0.92 | 0.24 | 0.18 | 0.29 | 0.14 | 0.15 | 0.71 | 0.27 | 0.19 | 0.15 | 0.23 | 0.35 |
| Altrose | 0.92 | 1.21 | 0.83 | 1.05 | 1.48 | 0.91 | 0.43 | 0.26 | 0.54 | 0.29 | 2.12 | 0.55 | 0.35 | 0.32 | 0.43 | 0.68 |
| Ascorbic acid | 0.70 | 0.78 | 0.98 | 1.55 | 1.24 | 1.21 | 0.46 | 0.56 | 0.47 | 0.35 | 3.19 | 2.42 | 0.65 | 0.51 | 0.41 | 0.83 |
| Asparagine | 0.75 | 0.92 | 1.41 | 0.92 | 1.05 | 0.51 | 0.55 | 0.58 | 0.41 | 0.40 | 0.87 | 0.57 | 0.62 | 0.41 | 0.35 | 0.73 |
| Aspartic acid | 0.65 | 0.83 | 1.46 | 1.06 | 1.06 | 0.54 | 0.72 | 0.86 | 0.32 | 0.43 | 1.48 | 0.79 | 0.76 | 0.61 | 0.58 | 0.86 |
| Butyric acid, 4-amino- | 0.62 | 0.71 | 1.84 | 0.83 | 1.91 | 0.33 | 0.53 | 0.58 | 0.26 | 0.20 | 1.90 | 0.73 | 0.59 | 0.24 | 0.30 | 0.77 |
| Citric acid | 0.46 | 0.65 | 1.06 | 1.83 | 0.58 | 0.35 | 0.46 | 0.35 | 0.15 | 0.19 | 0.40 | 0.22 | 0.24 | 0.20 | 0.27 | 0.44 |
| Dodecanoic acid | 0.88 | 0.96 | 1.25 | 0.91 | 1.18 | 0.58 | 0.43 | 0.46 | 0.45 | 0.20 | 0.92 | 0.63 | 0.36 | 0.13 | 0.22 | 0.40 |
| Fructose | 0.91 | 1.01 | 1.11 | 0.97 | 2.40 | 1.13 | 0.64 | 0.38 | 0.67 | 0.36 | 4.03 | 0.98 | 0.67 | 0.57 | 0.69 | 1.52 |
| Fucose | 0.82 | 0.91 | 1.16 | 1.11 | 1.13 | 0.56 | 0.40 | 0.42 | 0.48 | 0.29 | 1.06 | 0.55 | 0.35 | 0.22 | 0.20 | 0.47 |
| Fumaric acid | 1.05 | 0.49 | 1.13 | 1.33 | 0.60 | 0.11 | 0.14 | 0.27 | 0.22 | 0.11 | 0.51 | 0.19 | 0.17 | 0.08 | 0.28 | 0.19 |
| Gluconic acid-1,5-lactone | 0.96 | 1.00 | 1.03 | 1.02 | 1.75 | 1.05 | 0.90 | 0.91 | 0.78 | 0.51 | 2.61 | 1.23 | 0.79 | 0.57 | 0.45 | 1.00 |
| Glucose | 0.92 | 1.21 | 0.83 | 1.05 | 1.48 | 0.91 | 0.43 | 0.26 | 0.54 | 0.29 | 2.12 | 0.55 | 0.35 | 0.32 | 0.43 | 0.68 |
| Glutamic acid | 0.90 | 0.57 | 1.67 | 0.86 | 0.39 | 0.35 | 0.57 | 0.43 | 0.13 | 0.28 | 1.59 | 0.46 | 0.36 | 0.39 | 0.37 | 0.96 |
| Glutamine | 1.07 | 0.43 | 1.69 | 0.81 | 2.18 | 0.47 | 0.49 | 1.24 | 0.20 | 0.60 | 2.08 | 0.70 | 0.67 | 0.42 | 0.75 | 1.43 |
| Glyceric acid | 0.84 | 1.10 | 0.92 | 1.14 | 6.52 | 2.85 | 3.21 | 3.64 | 1.73 | 1.88 | 10.18 | 3.50 | 2.54 | 2.08 | 1.80 | 4.56 |
| Glycerol | 0.60 | 0.99 | 1.37 | 1.04 | 0.89 | 0.29 | 0.45 | 0.34 | 0.25 | 0.22 | 0.94 | 0.61 | 0.50 | 0.23 | 0.24 | 0.46 |
| Glycine | 0.82 | 0.95 | 1.10 | 1.13 | 1.95 | 2.21 | 1.70 | 1.25 | 1.58 | 1.95 | 5.24 | 3.01 | 1.97 | 1.66 | 1.54 | 3.02 |
| Hexadecenoic acid | 0.88 | 1.08 | 1.19 | 0.85 | 0.77 | 0.44 | 0.52 | 0.47 | 0.36 | 0.27 | 0.99 | 0.51 | 0.57 | 0.23 | 0.31 | 0.57 |
| Inositol, myo- | 1.08 | 0.91 | 0.89 | 1.12 | 1.14 | 0.70 | 0.46 | 0.48 | 0.46 | 0.33 | 1.18 | 0.51 | 0.35 | 0.29 | 0.23 | 0.54 |
| Isocitric acid | 0.88 | 1.20 | 0.81 | 1.11 | 1.37 | 0.96 | 0.39 | 0.26 | 0.56 | 0.30 | 2.10 | 0.54 | 0.33 | 0.30 | 0.39 | 0.66 |
| Isoleucine | 0.66 | 0.61 | 1.88 | 0.85 | 3.87 | 0.51 | 0.76 | 0.66 | 0.41 | 0.26 | 2.54 | 1.19 | 0.57 | 0.43 | 0.57 | 1.06 |
| Leucine | 1.03 | 1.06 | 1.12 | 0.80 | 1.04 | 0.27 | 0.73 | 0.31 | 0.31 | 0.16 | 0.65 | 0.41 | 0.65 | 0.28 | 0.29 | 0.44 |
| Lysine | 0.92 | 0.64 | 1.64 | 0.80 | 2.00 | 0.48 | 0.72 | 0.55 | 0.37 | 0.34 | 2.30 | 1.10 | 0.45 | 0.46 | 0.53 | 1.08 |
| Malic acid | 0.79 | 0.55 | 1.43 | 1.22 | 2.40 | 0.70 | 0.61 | 1.75 | 0.47 | 0.54 | 1.89 | 0.81 | 0.79 | 0.58 | 1.17 | 1.05 |
| Mannitol | 0.93 | 1.02 | 1.04 | 1.00 | 2.39 | 1.16 | 0.68 | 0.39 | 0.66 | 0.34 | 4.27 | 1.04 | 0.70 | 0.56 | 0.66 | 1.52 |
| Mannose | 0.70 | 0.56 | 0.77 | 1.97 | 1.38 | 1.02 | 0.58 | 0.51 | 0.75 | 0.34 | 3.04 | 1.01 | 0.45 | 0.40 | 0.35 | 1.39 |
| Nicotinic acid | 0.73 | 1.11 | 1.42 | 0.74 | 2.30 | 0.56 | 0.88 | 1.17 | 0.51 | 0.51 | 2.09 | 1.03 | 0.87 | 0.59 | 0.57 | 1.33 |
| Octadecanoic acid | 0.87 | 1.05 | 1.31 | 0.77 | 0.66 | 0.42 | 0.51 | 0.41 | 0.31 | 0.24 | 0.85 | 0.38 | 0.56 | 0.19 | 0.27 | 0.54 |
| Phenylalanine | 0.76 | 0.76 | 1.70 | 0.77 | 2.15 | 0.29 | 0.44 | 0.30 | 0.28 | 0.15 | 1.95 | 0.83 | 0.39 | 0.31 | 0.40 | 0.78 |
| Proline | 1.07 | 0.82 | 0.78 | 1.34 | 0.60 | 0.70 | 0.48 | 0.41 | 0.35 | 0.20 | 0.50 | 0.46 | 0.30 | 0.19 | 0.70 | 0.35 |
| Putrescine | 0.81 | 1.20 | 1.09 | 0.90 | 1.07 | 0.70 | 0.82 | 0.56 | 0.41 | 0.27 | 1.25 | 0.65 | 0.63 | 0.40 | 0.38 | 0.82 |
| Pyridine, 2-hydroxy- | 0.99 | 0.78 | 1.14 | 1.09 | 1.44 | 0.71 | 0.84 | 0.39 | 0.30 | 0.39 | 1.84 | 0.86 | 0.81 | 0.25 | 0.33 | 0.63 |
| Pyroglutamic acid | 0.73 | 0.77 | 1.62 | 0.88 | 0.83 | 0.24 | 0.56 | 0.62 | 0.25 | 0.38 | 1.75 | 0.64 | 0.51 | 0.44 | 0.59 | 0.95 |
| Ribose | 0.97 | 0.99 | 1.03 | 1.01 | 0.93 | 1.09 | 1.19 | 0.96 | 0.69 | 1.02 | 1.09 | 1.04 | 1.22 | 0.91 | 0.68 | 1.01 |
| Ribose-5-phosphate | 0.90 | 1.18 | 0.94 | 0.98 | 1.94 | 0.81 | 0.72 | 1.22 | 0.55 | 0.42 | 2.53 | 1.14 | 0.52 | 0.35 | 0.27 | 0.99 |
| Serine | 0.67 | 0.80 | 1.65 | 0.88 | 4.45 | 4.31 | 4.12 | 1.97 | 3.12 | 2.82 | 12.25 | 4.63 | 3.57 | 4.23 | 3.09 | 6.17 |
| Spermidine | 1.19 | 1.00 | 0.85 | 0.95 | 0.64 | 0.43 | 0.21 | 0.18 | 0.17 | 0.12 | 0.43 | 0.22 | 0.15 | 0.06 | 0.05 | 0.13 |
| Succinic acid | 0.95 | 0.76 | 1.29 | 1.00 | 1.87 | 0.64 | 0.65 | 0.41 | 0.56 | 0.37 | 2.26 | 0.48 | 0.70 | 0.40 | 0.69 | 1.17 |
| Sucrose | 0.90 | 0.76 | 1.35 | 0.98 | 2.32 | 0.72 | 0.50 | 0.57 | 0.45 | 0.35 | 1.80 | 0.58 | 0.51 | 0.47 | 0.52 | 0.78 |
| Tagatose | 0.92 | 1.02 | 0.99 | 1.07 | 0.80 | 0.39 | 0.51 | 0.36 | 0.33 | 0.23 | 1.11 | 0.52 | 0.60 | 0.19 | 0.31 | 0.47 |
| Tetracosanoic acid methyl ester | 0.94 | 0.97 | 1.01 | 1.08 | 0.82 | 0.34 | 0.51 | 0.37 | 0.32 | 0.24 | 1.16 | 0.51 | 0.69 | 0.21 | 0.33 | 0.50 |
| Threonic acid | 0.65 | 0.86 | 1.27 | 1.22 | 1.36 | 0.46 | 0.60 | 0.66 | 0.47 | 0.41 | 1.33 | 0.71 | 0.47 | 0.41 | 0.41 | 0.67 |
| Tyramine | 0.94 | 1.08 | 1.11 | 0.86 | 0.60 | 0.49 | 0.51 | 0.47 | 0.39 | 0.37 | 1.14 | 0.50 | 0.58 | 0.32 | 0.30 | 0.50 |
| Tyrosine | 0.78 | 0.66 | 1.47 | 1.09 | 1.65 | 0.33 | 0.62 | 0.33 | 0.24 | 0.24 | 2.12 | 1.23 | 0.62 | 0.59 | 0.88 | 1.01 |
| Valine | 0.71 | 0.64 | 1.68 | 0.97 | 1.80 | 0.30 | 0.38 | 0.41 | 0.27 | 0.17 | 1.42 | 0.64 | 0.31 | 0.27 | 0.33 | 0.58 |
|  | *pgam-pgam1/2-1* | *pgam-pgam1/2-2* | *pgam-pgam1/2-3* | *pgam-pgam1/2-4* | *nA- pgam1/2-1* | *nA- pgam1/2-2* | *nA- pgam1/2-3* | *nA- pgam1/2-4* | *pgam1/2-1* | *pgam1/2-2* | *pgam1/2-3* | *pgam1/2-4* | *sdmA-pgam1/2-1* | *sdmA-pgam1/2-2* | *sdmA-pgam1/2-3* | *sdmA-pgam1/2-4* |
| Ribitol | 1.02 | 1.00 | 1.00 | 0.99 | 0.98 | 1.47 | 0.84 | 0.86 | 0.85 | 1.06 | 1.25 | 1.25 | 1.21 | 1.25 | 1.04 | 1.05 |
| Aconitic acid, cis- | 1.00 | 1.04 | 0.98 | 0.89 | 0.95 | 1.21 | 0.74 | 0.72 | 0.18 | 0.90 | 0.27 | 0.28 | 0.17 | 0.20 | 0.14 | 0.49 |
| Alanine | 1.22 | 0.96 | 0.68 | 1.61 | 1.19 | 0.74 | 0.92 | 0.74 | 0.08 | 0.41 | 0.25 | 0.29 | 0.21 | 0.09 | 0.17 | 0.22 |
| Altrose | 1.55 | 1.38 | 0.59 | 1.43 | 0.70 | 0.56 | 1.01 | 0.43 | 0.29 | 0.75 | 0.26 | 0.46 | 0.12 | 0.21 | 0.07 | 0.31 |
| Ascorbic acid | 1.12 | 1.45 | 1.51 | 1.31 | 1.50 | 0.95 | 2.30 | 1.22 | 0.20 | 1.86 | 1.57 | 3.10 | 0.22 | 1.33 | 0.36 | 0.58 |
| Asparagine | 0.60 | 0.80 | 0.43 | 1.03 | 1.03 | 1.33 | 1.17 | 1.13 | 0.25 | 1.03 | 0.57 | 0.67 | 0.20 | 0.18 | 0.26 | 0.71 |
| Aspartic acid | 0.38 | 0.55 | 0.92 | 0.86 | 1.20 | 1.50 | 1.79 | 1.72 | 0.42 | 1.29 | 0.85 | 1.22 | 0.25 | 1.14 | 0.26 | 0.39 |
| Butyric acid, 4-amino- | 1.02 | 0.80 | 0.73 | 1.02 | 0.82 | 0.90 | 0.55 | 0.53 | 0.22 | 0.85 | 0.31 | 0.35 | 0.20 | 0.24 | 0.17 | 0.42 |
| Citric acid | 0.21 | 0.23 | 0.71 | 0.62 | 0.91 | 0.45 | 0.73 | 0.56 | 0.22 | 0.68 | 1.34 | 2.34 | 0.18 | 3.62 | 0.27 | 0.23 |
| Dodecanoic acid | 0.97 | 0.80 | 1.16 | 1.04 | 0.61 | 1.12 | 1.33 | 1.14 | 0.29 | 1.18 | 0.36 | 0.43 | 0.11 | 0.19 | 0.12 | 0.40 |
| Fructose | 1.12 | 1.41 | 0.65 | 1.09 | 0.55 | 0.36 | 1.66 | 0.27 | 0.28 | 0.77 | 0.37 | 0.75 | 0.18 | 0.32 | 0.05 | 0.33 |
| Fucose | 0.83 | 0.87 | 0.84 | 0.89 | 0.75 | 0.80 | 1.03 | 0.96 | 0.19 | 1.03 | 0.35 | 0.51 | 0.17 | 0.15 | 0.15 | 0.31 |
| Fumaric acid | 0.45 | 1.08 | 0.82 | 0.41 | 1.16 | 0.67 | 0.65 | 0.72 | 0.10 | 0.27 | 0.10 | 0.21 | 0.32 | 0.14 | 0.16 | 0.13 |
| Gluconic acid-1,5-lactone | 1.13 | 1.12 | 0.78 | 0.80 | 1.53 | 1.57 | 1.25 | 1.70 | 0.51 | 2.13 | 0.50 | 1.43 | 0.29 | 0.41 | 0.41 | 0.71 |
| Glucose | 1.55 | 1.38 | 0.59 | 1.43 | 0.70 | 0.56 | 1.01 | 0.43 | 0.29 | 0.75 | 0.26 | 0.46 | 0.12 | 0.21 | 0.07 | 0.31 |
| Glutamic acid | 0.81 | 0.91 | 1.28 | 0.88 | 0.81 | 0.58 | 0.78 | 0.65 | 0.28 | 1.09 | 0.71 | 0.93 | 0.12 | 0.65 | 0.13 | 0.27 |
| Glutamine | 0.83 | 1.18 | 1.01 | 0.87 | 2.23 | 2.00 | 1.97 | 1.80 | 0.62 | 2.85 | 1.19 | 1.78 | 0.44 | 0.57 | 0.31 | 0.31 |
| Glyceric acid | 1.04 | 0.71 | 0.79 | 1.40 | 1.85 | 2.25 | 2.02 | 2.10 | 1.61 | 6.73 | 2.81 | 2.69 | 1.12 | 2.30 | 1.50 | 2.07 |
| Glycerol | 1.83 | 0.54 | 0.53 | 0.88 | 0.52 | 0.81 | 0.50 | 0.53 | 0.12 | 0.59 | 0.18 | 0.18 | 0.13 | 0.25 | 0.15 | 0.33 |
| Glycine | 1.83 | 2.37 | 3.14 | 1.84 | 1.06 | 1.40 | 2.16 | 0.79 | 0.91 | 3.44 | 1.64 | 1.36 | 0.49 | 1.24 | 1.28 | 1.70 |
| Hexadecenoic acid | 1.13 | 0.87 | 1.09 | 0.94 | 0.91 | 1.17 | 0.84 | 0.69 | 0.25 | 1.10 | 0.28 | 0.36 | 0.19 | 0.23 | 0.17 | 0.55 |
| Inositol, myo- | 0.85 | 0.98 | 0.97 | 0.89 | 0.96 | 0.81 | 0.89 | 0.71 | 0.22 | 0.98 | 0.34 | 0.72 | 0.27 | 0.23 | 0.26 | 0.45 |
| Isocitric acid | 1.48 | 1.35 | 0.55 | 1.39 | 0.72 | 0.55 | 1.04 | 0.40 | 0.30 | 0.75 | 0.29 | 0.53 | 0.12 | 0.24 | 0.07 | 0.30 |
| Isoleucine | 1.02 | 0.63 | 0.55 | 1.03 | 0.67 | 0.56 | 0.83 | 0.64 | 0.21 | 1.23 | 0.52 | 0.64 | 0.19 | 0.22 | 0.16 | 0.43 |
| Leucine | 0.33 | 0.45 | 0.71 | 0.62 | 0.46 | 1.36 | 0.97 | 0.82 | 0.10 | 0.93 | 0.18 | 0.19 | 0.19 | 0.12 | 0.18 | 0.28 |
| Lysine | 1.11 | 0.60 | 0.63 | 0.60 | 0.81 | 0.82 | 1.00 | 0.70 | 0.28 | 1.16 | 0.40 | 0.69 | 0.10 | 0.19 | 0.13 | 0.39 |
| Malic acid | 0.39 | 0.76 | 0.86 | 0.83 | 1.58 | 1.55 | 2.21 | 1.65 | 0.66 | 2.06 | 1.28 | 1.25 | 0.87 | 0.96 | 0.87 | 0.59 |
| Mannitol | 1.10 | 1.50 | 0.69 | 1.04 | 0.56 | 0.39 | 1.66 | 0.27 | 0.30 | 0.79 | 0.37 | 0.73 | 0.18 | 0.37 | 0.05 | 0.34 |
| Mannose | 2.06 | 0.80 | 1.10 | 0.97 | 0.90 | 0.81 | 1.49 | 0.87 | 0.30 | 1.28 | 0.39 | 0.66 | 0.22 | 0.49 | 0.15 | 0.42 |
| Nicotinic acid | 0.69 | 0.77 | 0.70 | 1.65 | 0.79 | 1.38 | 1.60 | 1.60 | 0.31 | 1.82 | 0.48 | 0.66 | 0.28 | 0.35 | 0.38 | 0.38 |
| Octadecanoic acid | 1.13 | 0.77 | 1.32 | 0.82 | 0.89 | 1.04 | 0.99 | 0.68 | 0.23 | 0.96 | 0.27 | 0.34 | 0.14 | 0.23 | 0.15 | 0.54 |
| Phenylalanine | 0.82 | 0.60 | 0.63 | 1.05 | 0.51 | 0.25 | 0.68 | 0.44 | 0.15 | 0.85 | 0.30 | 0.43 | 0.13 | 0.15 | 0.07 | 0.25 |
| Proline | 1.39 | 1.18 | 0.96 | 2.06 | 1.19 | 0.63 | 0.52 | 0.66 | 0.10 | 0.54 | 0.20 | 0.49 | 0.34 | 0.24 | 0.19 | 0.40 |
| Putrescine | 1.40 | 1.13 | 0.97 | 1.86 | 1.21 | 0.75 | 1.01 | 0.95 | 0.22 | 1.44 | 0.67 | 0.82 | 0.21 | 0.21 | 0.21 | 0.76 |
| Pyridine, 2-hydroxy- | 1.30 | 0.99 | 0.66 | 1.26 | 0.85 | 1.53 | 0.56 | 0.92 | 0.19 | 0.87 | 0.23 | 0.34 | 0.26 | 0.29 | 0.34 | 0.68 |
| Pyroglutamic acid | 0.48 | 0.48 | 0.58 | 0.53 | 0.65 | 0.98 | 0.89 | 1.14 | 0.27 | 1.06 | 0.32 | 0.54 | 0.43 | 0.32 | 0.29 | 0.27 |
| Ribose | 1.01 | 0.98 | 0.98 | 1.00 | 0.95 | 1.43 | 0.81 | 0.84 | 0.84 | 1.03 | 1.23 | 1.22 | 1.18 | 1.24 | 1.03 | 1.06 |
| Ribose-5-phosphate | 1.39 | 0.89 | 0.54 | 0.72 | 1.19 | 1.66 | 0.77 | 1.86 | 0.42 | 2.35 | 0.37 | 1.61 | 0.22 | 0.12 | 0.35 | 0.50 |
| Serine | 1.12 | 0.71 | 0.68 | 1.32 | 2.00 | 2.39 | 3.12 | 3.23 | 1.36 | 7.09 | 2.47 | 4.18 | 0.70 | 2.12 | 0.54 | 2.63 |
| Spermidine | 1.21 | 1.25 | 0.91 | 1.18 | 0.90 | 0.77 | 0.81 | 0.55 | 0.06 | 0.51 | 0.16 | 0.28 | 0.05 | 0.12 | 0.10 | 0.29 |
| Succinic acid | 1.44 | 0.75 | 0.76 | 1.15 | 0.64 | 0.82 | 0.92 | 0.85 | 0.19 | 0.96 | 0.35 | 0.49 | 0.13 | 0.37 | 0.17 | 0.57 |
| Sucrose | 1.12 | 0.75 | 0.66 | 1.09 | 1.07 | 0.92 | 1.10 | 1.15 | 0.24 | 1.21 | 0.52 | 0.62 | 0.34 | 0.32 | 0.31 | 0.42 |
| Tagatose | 0.98 | 0.92 | 0.88 | 0.97 | 0.88 | 1.15 | 0.76 | 0.71 | 0.17 | 0.88 | 0.25 | 0.26 | 0.17 | 0.19 | 0.16 | 0.46 |
| Tetracosanoic acid methyl ester | 0.99 | 0.97 | 0.93 | 1.00 | 0.83 | 1.07 | 0.64 | 0.61 | 0.16 | 0.80 | 0.22 | 0.21 | 0.18 | 0.19 | 0.17 | 0.47 |
| Threonic acid | 0.59 | 0.74 | 0.88 | 1.08 | 0.70 | 0.93 | 1.01 | 0.84 | 0.34 | 1.10 | 0.72 | 0.92 | 0.31 | 0.59 | 0.31 | 0.35 |
| Tyramine | 0.83 | 1.08 | 0.96 | 0.93 | 0.81 | 0.66 | 0.47 | 0.50 | 0.23 | 1.28 | 0.36 | 0.52 | 0.18 | 0.23 | 0.24 | 0.58 |
| Tyrosine | 0.94 | 0.54 | 0.71 | 1.01 | 0.77 | 0.93 | 0.50 | 0.44 | 0.21 | 1.00 | 0.49 | 0.65 | 0.08 | 0.27 | 0.14 | 0.46 |
| Valine | 1.05 | 0.69 | 0.57 | 1.26 | 0.57 | 0.59 | 0.72 | 0.52 | 0.12 | 0.70 | 0.33 | 0.35 | 0.16 | 0.12 | 0.16 | 0.26 |

| Supplementary table7 Standard used for the GC-MS | | | |
| --- | --- | --- | --- |
| **no** | **Compound** |  |  |
| **1** | **Putrescine dihydrochloride** |  |  |
| **2** | **Histidine** |  |  |
| **3** | **beta-Alanine** |  |  |
| **4** | **Glucose** |  |  |
| **5** | **Serine** |  |  |
| **6** | **Galactinol Dihydrate** |  |  |
| **7** | **Ornithine monohydrochloride, L-** |  |  |
| **8** | **L-Alanine** |  |  |
| **9** | **Malic acid** |  |  |
| **10** | **Citric acid** |  |  |
| **11** | **myo-Inositol** |  |  |
| **12** | **Arginine** |  |  |
| **13** | **Leucine** |  |  |
| **14** | **Sucrose** |  |  |
| **15** | **Valine** |  |  |
| **16** | **Tyrosine** |  |  |
| **17** | **Erythritol** |  |  |
| **18** | **Fumaric acid** |  |  |
| **19** | **Glutamine** |  |  |
| **20** | **Glycerol** |  |  |
| **21** | **Glycine** |  |  |
| **22** | **Aspartic acid** |  |  |
| **23** | **Isoleucine** |  |  |
| **24** | **Fructose** |  |  |
| **25** | **Proline** |  |  |
| **26** | **Glutamic acid** |  |  |
| **27** | **Succinic acid** |  |  |
| **28** | **Asparagine** |  |  |
| **29** | **D-(+)-Raffinose** |  |  |
| **30** | **Lysine** |  |  |
| **31** | **Trehalose dihydrate, D-(+)-** |  |  |
| **32** | **Xylose** |  |  |
| **33** | **Butyric acid, 4-amino-** |  |  |
| **34** | **Methionine** |  |  |
| **35** | **Phenylalanine** |  |  |
| **36** | **Phosphoric Acid** |  |  |
| **37** | **Dehydroascorbic acid** |  |  |
| **38** | **Pyruvic acid sodium salt** |  |  |
| **39** | **Glutaric acid, 2-oxo-** |  |  |
| **40** | **Tryptophan** |  |  |
| **41** | **Glycerol 3-P** |  |  |
